# ‘Forest malaria’ in Myanmar? Tracking transmission landscapes in a diversity of environments

**DOI:** 10.1186/s13071-023-05915-w

**Published:** 2023-09-12

**Authors:** Eva Legendre, Florian Girond, Vincent Herbreteau, Sokeang Hoeun, Stanislas Rebaudet, Aung Myint Thu, Jade Dean Rae, Laurent Lehot, Sokhna Dieng, Gilles Delmas, François Nosten, Jean Gaudart, Jordi Landier

**Affiliations:** 1 Aix Marseille Univ, IRD, INSERM, SESSTIM, ISSPAM, 27 boulevard Jean Moulin, 13005 Marseille, France; 2 Institut de Recherche pour le Développement, UMR 228 Espace-Dev (IRD, UA, UG, UM, UR), Phnom Penh, Cambodia; 3 Institut Pasteur du Cambodge, Phnom Penh, Cambodia; 4 Hôpital Européen Marseille, Marseille, France; 5 Shoklo Malaria Research Unit, Mahidol Oxford Tropical Medicine Research Unit, Mae Sot, Thailand; 6 Mahidol-Oxford Tropical Medicine Research Unit, Faculty of Tropical Medicine, Mahidol University, Bangkok, Thailand; 7 Centre for Tropical Medicine and Global Health, Nuffield Department of Medicine Research Building, University of Oxford, Old Road campus, Oxford, UK; 8 Aix Marseille Univ, IRD, INSERM, AP-HM, SESSTIM, La Timone Hospital, BioSTIC, Biostatistics and ICT, Marseille, France

**Keywords:** Malaria, Environment, Stratification, Mapping, Eco-epidemiological zones, Greater Mekong Subregion

## Abstract

**Background:**

In the Greater Mekong Subregion, case–control studies and national-level analyses have shown an association between malaria transmission and forest activities. The term ‘forest malaria’ hides the diversity of ecosystems in the GMS, which likely do not share a uniform malaria risk. To reach malaria elimination goals, it is crucial to document accurately (both spatially and temporally) the influence of environmental factors on malaria to improve resource allocation and policy planning within given areas. The aim of this ecological study is to characterize the association between malaria dynamics and detailed ecological environments determined at village level over a period of several years in Kayin State, Myanmar.

**Methods:**

We characterized malaria incidence profiles at village scale based on intra- and inter-annual variations in amplitude, seasonality, and trend over 4 years (2016–2020). Environment was described independently of village localization by overlaying a 2-km hexagonal grid over the region. Specifically, hierarchical classification on principal components, using remote sensing data of high spatial resolution, was used to assign a landscape and a climate type to each grid cell. We used conditional inference trees and random forests to study the association between the malaria incidence profile of each village, climate and landscape. Finally, we constructed eco-epidemiological zones to stratify and map malaria risk in the region by summarizing incidence and environment association information.

**Results:**

We identified a high diversity of landscapes (*n* = 19) corresponding to a gradient from pristine to highly anthropogenically modified landscapes. Within this diversity of landscapes, only three were associated with malaria-affected profiles. These landscapes were composed of a mosaic of dense and sparse forest fragmented by small agricultural patches. A single climate with moderate rainfall and a temperature range suitable for mosquito presence was also associated with malaria-affected profiles. Based on these environmental associations, we identified three eco-epidemiological zones marked by later persistence of *Plasmodium falciparum*, high *Plasmodium vivax* incidence after 2018, or a seasonality pattern in the rainy season.

**Conclusions:**

The term forest malaria covers a multitude of contexts of malaria persistence, dynamics and populations at risk. Intervention planning and surveillance could benefit from consideration of the diversity of landscapes to focus on those specifically associated with malaria transmission.

**Graphical Abstract:**

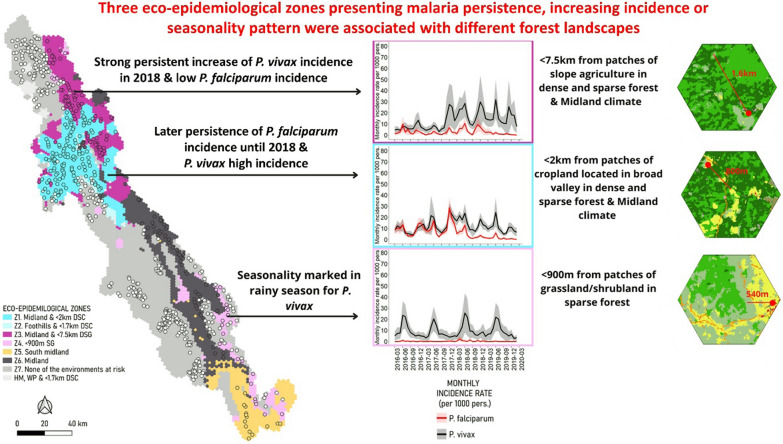

**Supplementary Information:**

The online version contains supplementary material available at 10.1186/s13071-023-05915-w.

## Background

The Greater Mekong Subregion (GMS) has made progress towards malaria elimination during the past decades. Between 2000 and 2016, the number of malaria cases declined sevenfold, dropping from approximately 2,400,000 cases to 325,000 due to improvements in early access to diagnosis and treatment, and targeted interventions for the most affected populations (i.e. migrant workers and displaced persons) [1–3]. These interventions were focused on *Plasmodium falciparum* to prevent the spread of multidrug-resistant parasite lineages [4]. The GMS now aims to eliminate all forms of malaria [1].

In the GMS, studies based on aggregated surveillance data associated malaria transmission with forested areas [5, 6]. Individual-scale studies consistently identified occupation-related risks factors, such as male sex, age over 15 years, or ‘going into the forest’ [7, 8]. ‘Forest malaria’ is often reported to be responsible for the remaining foci of transmission in the region, and is considered an obstacle to malaria elimination [9–11]. However, the term ‘forest’ is generic and covers a diversity of ecosystems in the GMS. Studies do not usually characterize the diversity and health of ‘forest environments’ to which individuals are exposed with respect to biodiversity, structure (size, fragmentation), seasonal variability, level of anthropogenic disturbance or conservation status.

Malaria transmission requires the presence of specific vectors and a sufficient density of human hosts over long enough periods of time to allow the parasite to complete its life cycle. It is therefore likely that the risk of malaria is not uniform across different types of forested ecosystems in the GMS. Activities related to these ecosystems (e.g. farming, rubber tapping, hunting, collecting forest products, logging, mining, patrolling) expose individuals to diverse environments, at different times.

As the incidence of malaria continues to decrease, the targeting of programmed or reactive interventions becomes increasingly relevant. According to the World Health Organization guidelines, to be most effective, interventions must be guided by malaria stratification, taking into account the intensity of transmission and the receptivity of ecosystems [9]. Malaria stratification is often only based on clinical incidence data, such as the annual parasite index. Receptivity of an ecosystem can be quantified through entomological studies, but these require significant human and financial resources. Ecosystems have to harbour a combination of suitable environment conditions for vector development and human presence to be receptive. The study of environments and associated human activities could therefore provide proxies [10]. It is crucial to determine accurately the influence of environmental factors on the risk of malaria transmission to improve stratification, resource allocation and effectiveness of elimination efforts.

Within the GMS, Myanmar accounts for 90% of malaria cases and remains the country with the highest malaria burden. In 2016, the countrywide incidence of malaria reached 0.45 cases per 1000 individuals at risk. However, Myanmar exhibits high regional and local spatial heterogeneity, with local foci of high malaria incidence persisting in mountainous-forest borderlands, such as Karen/Kayin State [1, 12]. In response to this heterogeneity, thorough malaria stratification is particularly relevant. We thus aimed to study and map the heterogeneity of malaria dynamics according to its relationship with environmental factors. We also aimed to improve malaria stratification by examining detailed descriptions of malaria incidence together with information on diverse environments in Karen/Kayin State.

## Methods

### Study design and setting

Karen/Kayin State is largely covered by the north–south-oriented, forested, Dawna range, which reaches an altitude of 2080 m above sea level. Settlements are located on the slopes and in the valleys (i.e. agricultural villages, refugee or military camps) of this mountain range. The density of villages is higher on the plain to the west of the mountains [11].

The Malaria Elimination Task Force (METF) program was initiated in 2014 in this region to eliminate artemisinin-resistant *P. falciparum*. We use the term ‘METF region’ to represent the area where the program was implemented, which covers four townships in Karen/Kayin State: Myawaddy, Kawkareik, Hlaingbwe and Hpapun [13]. This program provided access to early diagnosis and treatment through community-based malaria posts (MP) in 95% of the villages identified in the target region by 2016. Cases of fever were systematically tested by a *Plasmodium falciparum*-*Plasmodium vivax* rapid diagnostic test (SD Bioline Ag P.f./P.v.) at the MPs, which send weekly reports specifying the number of diagnosed cases, by age group and gender, of *P. falciparum* and *P. vivax* to METF [13]. In addition, mass drug administration (MDA) was conducted in villages where real-time quantitative polymerase chain reaction surveys detected high malaria prevalence [12, 13]. During the first year of the METF program (May 2014–April 2015), the incidence of *P. falciparum* was 39 cases per 1000 person-years, while the incidence of *P. vivax* was 63 cases per 1000 person-years [12]. Following 4 years of program implementation, there was a significant reduction in the incidence of *P. falciparum* and *P. vivax* in the four townships. Specifically, *P. falciparum* incidence decreased by 97% (1 case per 1000 person-years), and *P. vivax* incidence decreased by 71% (18.5 cases per 1000 person-years) in the period from May 2019 to April 2020.

The first aim of this ecological study was to describe ecological landscapes and climate diversity in Karen/Kayin State. We described forest landscapes at the METF region level. We then focused on a single township (corresponding to Hpapun/Mutraw administrative township; hereafter ‘Northern Township’) where malaria incidence was highest [12]. Second, we conducted an association study between village incidence profiles (for *P. falciparum* and *P. vivax*), MDA, climate and distance between villages and landscapes at the METF region scale and at the Northern Township scale. Based on this association study, we established a map of eco-epidemiological zones in the METF region, i.e. described climate and landscapes that likely drive local heterogeneity in malaria dynamics.

### Data

#### Outcome: malaria incidence profiles

This study was based on previous work that described malaria incidence heterogeneity in Karen/Kayin State from March 2016 to February 2020 for 662 villages [14]. Based on *P. falciparum* and *P. vivax* weekly incidence reported by MPs, villages sharing similar dynamics were grouped in *P. falciparum* and *P. vivax* ‘incidence profiles’. Incidence profiles were determined by partitioning around medoids clustering combined with dynamic time warping distance following functional time series smoothing. The methodology is described in this earlier study [14].

**Fig. 1:**
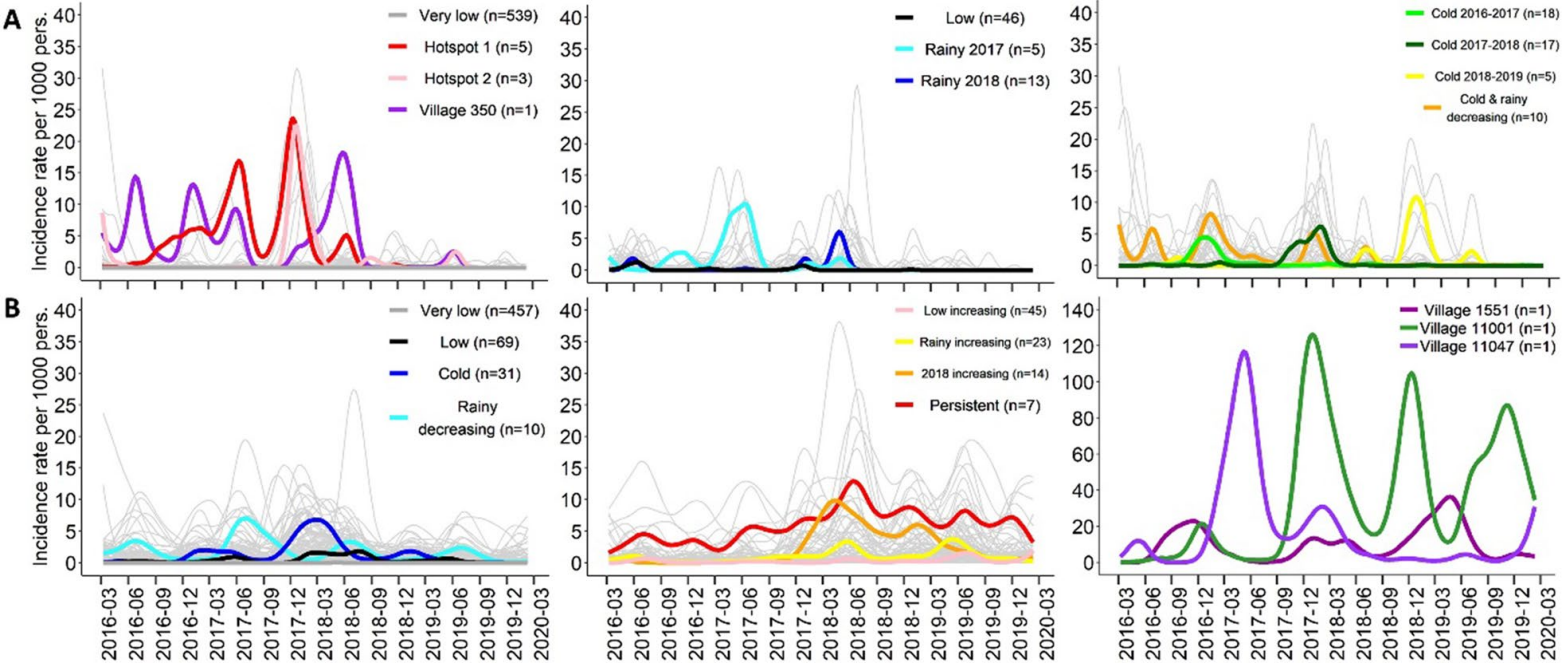
Malaria incidence profiles. **A** *Plasmodium falciparum* and **B** *Plasmodium vivax* incidence profiles of the 662 villages. Coloured lines correspond to the central village (medoid) of each cluster. Grey lines represent functional incidence rate of all villages included in the cluster across study period. This original figure presents data and results published previously [14]

Eleven *P. falciparum* and 11 *P. vivax* incidence profiles were identified in this previous work [14] and are used as the outcomes in the present study. *Plasmodium falciparum* incidence profiles distinguished villages by the amplitude of the incidence (Very low, Low vs. Hotspot) and the occurrence of seasonal sporadic annual peaks (Rainy 2017, Rainy 2018, Cold 2018–2019, etc.). *Plasmodium vivax* incidence profiles also separated villages according to incidence amplitude (Very low, Low, vs. Persistent), but also by intra-annual patterns with one or two annual peaks, their seasonality and tendency (Cold, Rainy increasing, Rainy decreasing, etc.) (Fig. 1). We named the *P. falciparum* or *P. vivax* incidence profiles that were different from the Very low clusters (i.e. that grouped the 10 other clusters) ‘malaria-affected incidence profiles’.

Malaria-affected incidence profiles (*n* = 123 for *P. falciparum* and *n* = 205 for *P. vivax*) were grouped in the Northern Township of Karen/Kayin State, and along the Thailand-Myanmar border for *P. vivax* (Additional file 1: Fig. S1) [14].

#### Environmental data

We described the climate and landscape over the entire region regardless of the location of the villages. To characterize environment systematically, a 2-km-wide hexagonal grid was overlaid over the study region and environmental raster data were extracted for each grid cell.

#### Climate

Meteorological information was obtained using Google Earth Engine and the rgee package to access MODIS data (MODIS/006/MOD11A2) for day and night land surface temperature (at about 1-km spatial resolution) and Climate Hazards Group InfraRed Precipitation with Station (CHIRPS) data (UCSB-CHG/CHIRPS/DAILY) for daily rainfall (at about 5.5-km spatial resolution) [15, 16]. The 2014–2020 time series (i.e. corresponding to the METF project period) obtained for each cell were averaged as monthly values and a principal component analysis (PCA) followed by a hierarchical ascendant clustering (HAC) was applied to identify cells sharing similar climates. Principal components explaining 99% of the variance were selected in the HAC, assuming that residual information was noise. Information on temperature for June, July and August was excluded from the PCA because of too many missing data points due to cloud cover during the rainy season. Information on rainfall for November, December, January, February and March, the cold dry season, was also excluded from the PCA because of the occurrence of rare, local, intense thunderstorms which could have influenced the PCA. Each village was assigned the climate of the cell where it was located.

#### Landscape

To characterize landscapes in detail, we produced a land use land cover (LULC) map for the entire METF target region based on Sentinel-2 satellite images from 2019 to 2020 using object-based image analysis with eCognition software. These images have high spatial (10–60 m) and temporal resolutions (~ 5 days). In order to validate the accuracy of the LULC, the METF field team described LULC of 300 sites which were used as ground truth data. We completed the coverage provided by these observations with 300 photo-interpreted points from Google Earth (which shows very high spatial resolution satellite images that are recent but cannot be precisely dated). We extracted elevation and slope data of the METF target region from the GMTED 2010 digital elevation model (7.5 arc seconds, 220 m) [17]. We used HAC on PCA results to define landscapes, including fragmentation indices from LULC classification, elevation, and slope for each grid cell (Additional file 1: Method S1). We produced two sets of landscape profiles: one covering the METF region, and one for the Northern Township. We excluded from the analysis rare landscapes that covered < 1% of the METF region or Northern Township. For every village, we calculated the distance to the nearest cell of each landscape. More details on this are provided in the supplementary materials (Additional file 1: Method S1).

#### Mass drug administration

MDA interventions were conducted in 69 villages as part of the METF program; these were carried out in 35 villages before March 2016 and in 34 villages during the study period (Additional file 1: Fig. S2). MDA may have influenced the malaria dynamics by inducing a transient decrease of *P. vivax* incidence and a long-term reduction of *P. falciparum* incidence [18, 19]. For *P. vivax*, we considered MDA campaigns undertaken during the study period (i.e. from March 2016 to February 2020). For *P. falciparum*, we considered MDA campaigns undertaken before the study period to distinguish villages that may have been at high risk before the study period. We did not consider the MDA during the study period for *P. falciparum* because it was implemented in villages with high prevalence, and thus was associated with high incidence [12].

### Statistical analysis

**Fig. 2:**
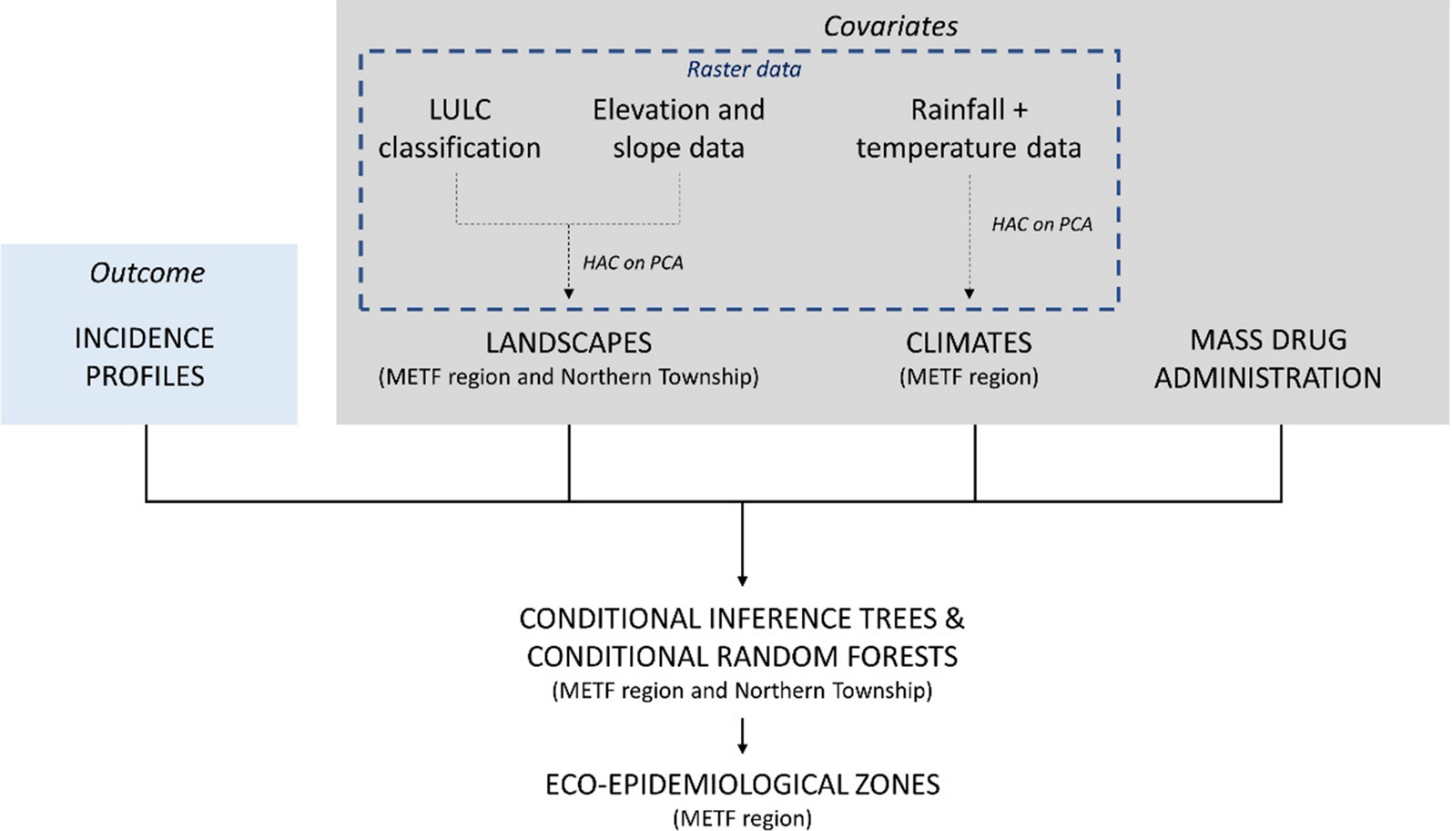
Schematic of the statistical plan. Hierarchical ascendant clustering (*HAC*) on principal components analysis (*PCA*) built up landscapes and climates based on land use land cover (*LULC*) or rainfall and temperature data. Conditional inference trees (CIT) and conditional random forests (CRF) realized at region and township scales explored associations between environmental factors and malaria incidence profiles. Eco-epidemiological zones were constructed based on climates and distance to landscapes significantly associated with *Plasmodium falciparum* and *Plasmodium vivax* incidence profiles in CIT and CRF. *METF* Malaria elimination task force

Figure 2 provides an overview of the statistical plan followed for *P. falciparum* and *P. vivax* data analysis. Analyses were conducted using R 4.0 and fda, TSclust, dtw, party, FactoMineR, and factoextra packages [20]. Maps were produced with QGIS 3.6.3 software [21].

#### Association between environmental factors and incidence profiles

We used conditional inference trees (CIT) and conditional random forests (CRF) to explore the association between *P. falciparum* and *P. vivax* incidence profiles, landscapes, climate, and MDA. CIT is a method similar to classification and regression tree (CART) that limits CART bias in covariate selection [22]. We used the following CIT parameters: c_quad_ test, distribution estimation by Monte Carlo method with 100,000 replications, 0.95 = 1—α as splitting criteria value of statistic test, and no minimum of node sample size. The CIT method provides an interpretable tree for covariate effects and interaction. We also used CRF to quantify covariate importance. We used the following CRF parameters: 10,000 trees, and number of covariates randomly selected for each tree equal to the minimum of the number of covariates included divided by 2 [22, 23].

Both mosquito dispersion and human mobility occur within a limited radius around the Karen villages included in this study [24, 25]. We applied a logarithmic transformation to the distance to landscape to give a heavier weight to landscapes within 1 km of the villages. Second, to limit the influence of environments beyond the range of reasonable occasional mobility, only landscapes at a median distance of ≤ 10 km from villages were considered. We conducted sensitivity analyses with 5 km and 15 km cut-offs to test the influence of variable selection on the robustness of the CIT and CRF results. These analyses were carried out separately at the METF-region and Northern-Township scales and for *P. falciparum* and *P. vivax*.

#### Definition of eco-epidemiological zones

Eco-epidemiological zones were defined based on variables significantly associated with *P. falciparum* and *P. vivax* incidence profiles obtained with CIT and confirmed by CRF. Specifically, we crossed CIT nodes regrouping malaria-affected incidence profiles and defined by their proximity to an environmental variable. Successive crossing tables of selected nodes defined eco-epidemiological zones as follows: 1/region vs. township scale for *P. falciparum*; 2/region vs. township scale for *P. vivax*; 3/*P. falciparum* vs. *P. vivax*.

## Results

### Climate description

**Fig. 3:**
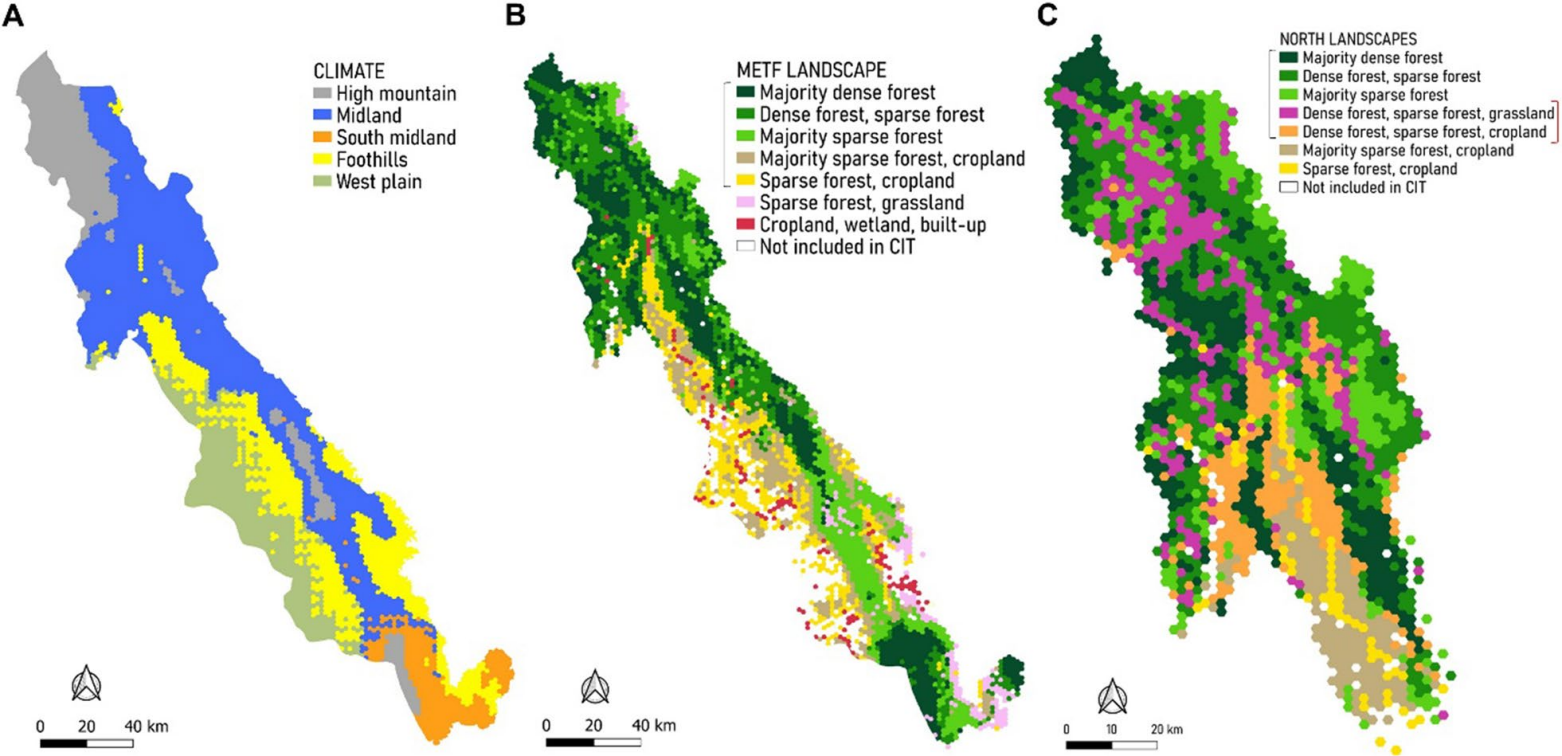
Climate and landscapes covariates. **A** Map of climates constructed at the METF region scale. **B** Map of identified landscapes that were included in the association analysis at the METF region scale. **C** Map of identified landscapes included in the association analysis when restricting the analysis to the Hpapun/Mutraw administrative township (Northern Township). Black braces indicate landscapes with a median distance of ≤ 10 km from villages. Red brace indicates landscapes identified only in the Northern Township. For abbreviations, see Fig. 2

The temperature and precipitation data defined five climatic zones in the METF region. These displayed a gradient of temperature (22–38 °C during the day and 15–25 °C at night, not modified by the period June–August) and precipitation (1290–3550 mm annually, without considering the period November–March). The High mountain climate had the lowest precipitation and temperature, the West plain climate the highest precipitation, and the Foothills climate the highest temperature. Villages with malaria-affected incidence profiles predominantly belonged to the Midland climate category: 92% (113/123) of villages affected by *P. falciparum* and 79% (161/205) by *P. vivax*. Midland climate presented moderate rainfall (1490 mm annually) and temperatures (24 °C on average) (Fig. 3a; Additional file 1: Fig. S3).

### Description and selection of landscapes

The final LULC classification produced from Sentinel-2 images from 2019 to 2020 includes the following 10 classes: dense forest, sparse forest, plantation, cropland, grass/shrubland, bare soil, wetland, road, water, built-up. The comparison with the observed field points indicated a satisfactory classification with a Cohen's kappa coefficient of 0.73 (0.66 with the ground truth points only, and 0.80 with the Google Earth observed points only). Confusion in the classification mainly arises between bare ground and crops, or between herbaceous areas and scattered woodland, which can be confounded depending on the date when the data were recorded. Weaker agreement with the data recorded in the field can be explained by the fact that these were often recorded at points on the side of roads, and therefore near other landscapes, and not at the centre of the landscapes they represented. In total, forested areas represent 76% of the surface area, and up to 78% if plantations and orchards are included. The Northern Township presented the most important surface area of dense forest (45%) compared to the rest of the METF region (12.5%). During the study period, no large-scale forest loss was detected in the METF region that drastically affected forest cover [26]. Croplands represented 3% of the METF region, but were widely heterogeneous between mountains and plains (from 0.5% in the north to 17% in the west) (Additional file 1: Fig. S4).

We merged water and wetland classes and excluded roads to obtain eight LULC classes. By analysing the percentage of surface covered by each class, fragmentation indices, slope and altitude, we could classify the entire region into 17 landscapes. These landscapes were diverse, with gradients of sparse and dense forest (up to 90% forest), altitude (averaging 17–1020 m above mean sea level) and with multiple combinations of cropland (0–96.5%), grassland/shrubland, or plantation (Additional file 1: Figs. S5, S6). Eleven landscapes were covered by > 50% forest. The type of landscape that was closest to most villages was dense and sparse forest only. The mean distance from villages to this landscape was 5 km (range 0–52 km) (Additional file 1: Fig. S7). Seven landscapes were retained for the analysis at the METF region scale; eight rare landscapes were excluded because they accounted for < 1% of the region’s surface; and two were excluded because they were too distant (median distance from village to landscape > 15 km) (Fig. 3b; Additional file 1: Fig S7A).

Likewise, seven landscapes were retained for analysis in the Northern Township (of the 13 identified, four rare and two distant landscapes were excluded) (Fig. 3c; Additional file 1: Fig. S7B). These seven landscapes were all characterized by > 50% sparse and dense forest cover. Four landscapes combined sparse forest with patches of cropland, grassland/shrubland or plantation. Two landscapes were specifically identified in the Northern Township: Dense forest, sparse forest, cropland (DSC) and Dense forest, sparse forest, grassland (DSG). These two landscapes mostly comprised dense and sparse forest (86% on average, of which 38% was dense and 48% sparse forest) with small patches of low vegetation, respectively, cropland (4% of the total surface, mean patch size = 4000 m^2^) or grassland/shrubland (8%; 4000 m^2^) (Additional file 1: Fig. S8). DSC and DSG landscapes corresponded to fragmented landscapes with two of the highest numbers of patches (*n* = 329 and 333, respectively) and the highest proportion of patches of sparse forest (40 and 32%, respectively) interspersed with cropland and grassland/shrubland.

### Association between malaria incidence profiles and climate

**Fig. 4:**
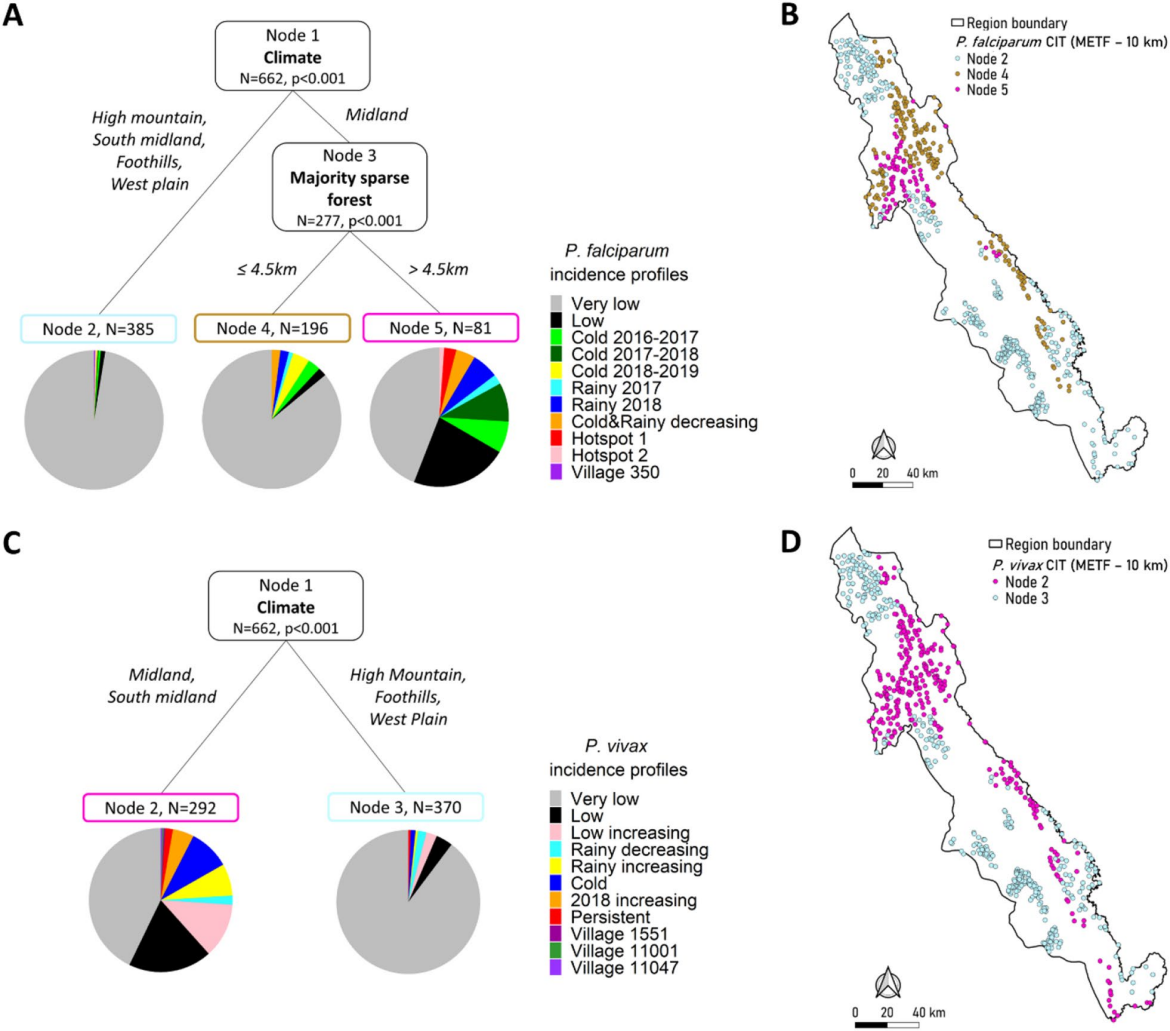
Association study between malaria incidence profiles and environment in the METF region by CITs. A 10-km cut-off was applied to select landscape covariates. **A** CIT of *Plasmodium falciparum* incidence profiles with respect to five landscapes, climates, and mass drug administration (MDA) before March 2016. **B** Location of villages depending on their nodes obtained with *P. falciparum* CIT. **C** CIT of *Plasmodium vivax* incidence profiles depending on five landscapes, climates, and MDA after February 2016. **D** Location of villages depending on their nodes obtained with *P. vivax* CIT. (In CIT, ‘node’ refers to a group of villages.) For other abbreviations, see Fig. 2

**Fig. 5:**
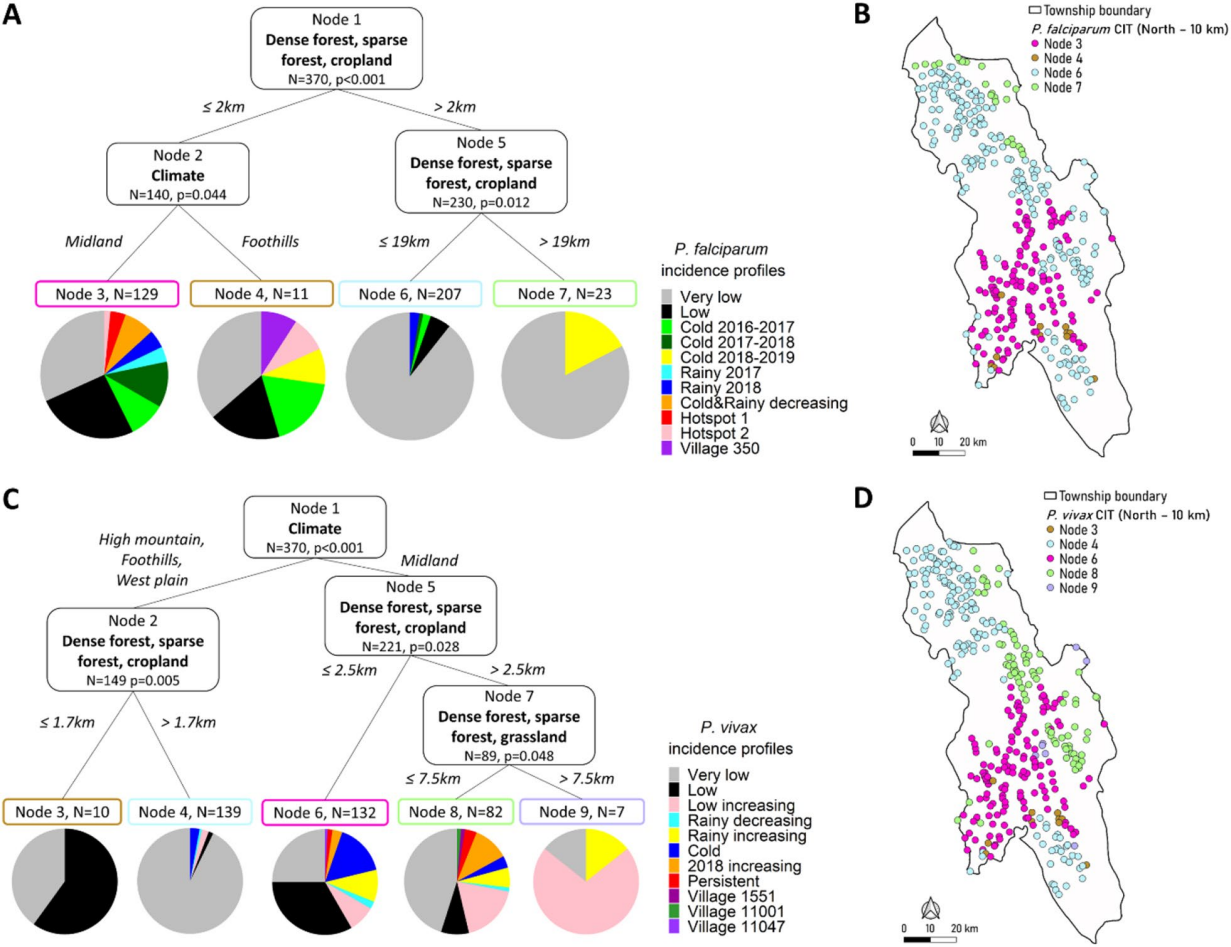
Association study between malaria incidence profiles and environment in Northern Township by CIT. A 10-km cut-off was applied to select landscape covariates. **A** CIT of *Plasmodium falciparum* incidence profiles depending on five landscapes, climates, and MDA before March 2016. **B** Location of villages depending on their nodes obtained with *P. falciparum* CIT. **C** CIT of *Plasmodium vivax* incidence profiles depending on five landscapes, climates, and MDA after February 2016. **D** Location of villages depending on their nodes obtained with *P. vivax* CIT. For abbreviations, see Figs. 2 and 4

CIT identified an association between Midland climate and malaria-affected *P. falciparum* and *P. vivax* incidence profiles in the METF region (*P* < 0.001) (Fig. 4). When limiting the analysis to the Northern Township, Midland climate was also associated with malaria-affected profiles for *P. falciparum* (*P* = 0.044) and for *P. vivax* (*P* < 0.001) (Fig. 5). CRF identified climate as the most important variable in the METF region and the second-most important variable in the Northern Township concerning *P. vivax*, which was consistent with the CIT results (Additional file 1: Figs. S9, S10). These results were consistent with those of the sensitivity analyses, with a 5-km or 15-km cut-off (Additional file 1: Figs. S7, S11, S12, S13, S14).

### Association between malaria incidence profiles and landscapes in the METF region

At the scale of the METF region, proximity to landscape was not associated with malaria-affected incidence profiles for *P. falciparum* and *P. vivax* with a 10 km cut-off (Fig. 4). Proximity to Sparse forest, grassland (SG) landscape (≤ 900 m; *P* < 0.001) was significantly associated with malaria-affected profiles for *P. vivax* in the analysis with a 15-km cut-off (Additional file 1: Fig. S11C). SG comprised 35% grass/shrubland, 34% sparse forest and 22% cropland (Additional file 1: Fig. S6). SG landscape was only selected when using the 15-km cut-off (Additional file 1: Fig. S7A). These villages were localized in the southeast area of the METF region (Additional file 1: Fig. S11D).

### Association between malaria incidence profiles and landscapes in the Northern Township

At the scale of the Northern Township, one main landscape was associated with malaria-affected incidence profiles for *P. falciparum* and *P. vivax*: DSC (Fig. 5). Proximity to DSC landscape was significantly associated with the highest proportion of malaria-affected incidence profiles for *P. falciparum* (≤ 2 km, *P* < 0.001) with a secondary climate-related division which distinguished only 11 villages located outside, but near, the Midland climate (*P* < 0.001) (Fig. 5a, b).

For *P. vivax*, a second landscape, DSG, was also associated with malaria-affected incidence profiles (≤ 7.5 km, *P* = 0.048) (Fig. 5c, d). Within Midland climate, DSC and DSG landscapes defined specific groups of villages with different proportions of *P. vivax* incidence profiles. The group located < 2.5 km from DSC included 80% of *P. vivax*-affected profiles. The group > 2.5 km away from DSC and located within 7.5 km of DSG landscape included 29 of the 82 villages (35%) with an increasing *P. vivax* incidence. CRF confirmed the importance of DSC and DSG landscapes. Subdivision by DSG landscape was significant according to first-order risk of 0.05 (*P* = 0.048), but was not confirmed by the sensitivity analysis (Additional file 1: Fig. S13C). All other results were consistent with the sensitivity analysis (Additional file 1: Figs. S13, S14).

### Identification and description of eco-epidemiological zones

**Fig. 6:**
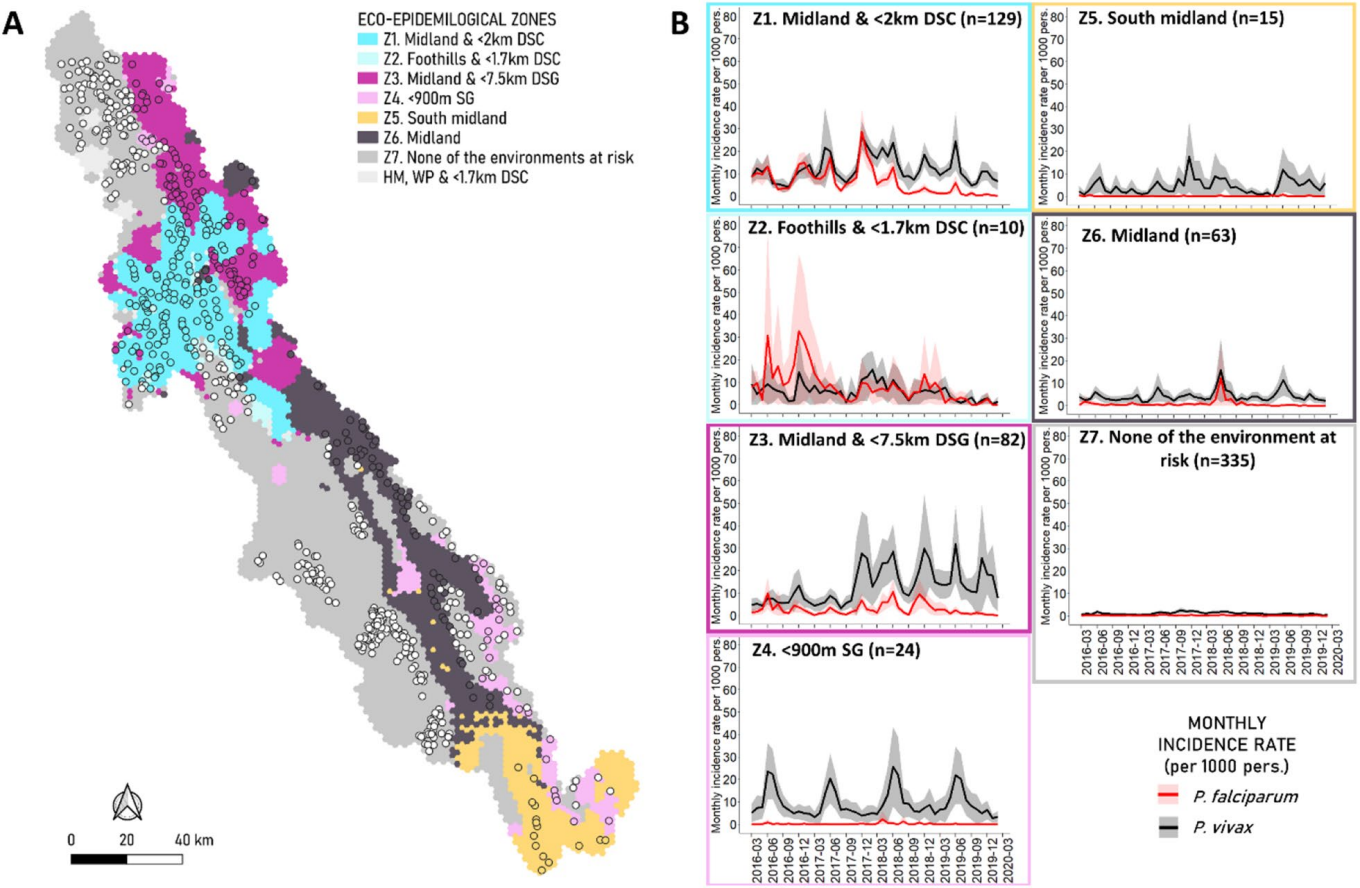
Description of eco-epidemiological zones. **A** Map of eco-epidemiological zones. Geographic localization of zones was determined at hexagon level according to their landscape and climate characteristics. **B** Description of the seven zones according to observed *Plasmodium falciparum* and *Plasmodium vivax* monthly incidence during the study period. *DSC* Dense forest, sparse forest, cropland landscape; *DSG* dense forest, sparse forest, grassland landscape; *SG* sparse forest, grassland landscape; *HM* high mountain climate; *WP* west plain climate; *Z* zone

Combining the climates and landscapes associated with malaria-affected incidence profiles (SG, DSC, DSG) produced seven eco-epidemiological zones (Fig. 6; see Additional file 1: Table S1 for details).

The combination of midland climate and proximity (< 2.5 km) of DSC landscape defined the highest environmental risk zone (zone 1) and included 129 villages of the Northern Township. *Plasmodium falciparum* incidence was the latest persistent until 2018. Cases occurred predominantly during the cold season (2.5 more villages with cold season profiles than rainy season profiles). *Plasmodium falciparum* incidence decreased after 2018 and *P. vivax* incidence remained stable, without clear seasonality. In addition, 10 adjacent villages sharing the same landscape and the same malaria dynamics were identified as a separate zone (zone 2) because of their warmer climate (Foothills). Zones 1 and 2 also stood out due to their high proportion of malaria cases among children under 5 years of age compared to other zones (40.8% vs. 19.5% mean for *P. vivax*, 20.2% vs. 12.3% mean for *P. falciparum*, respectively) (Additional file 1: Fig. S15).

The next zone, zone 3, included 82 villages of the Northern Township located in Midland climate, away from DSC (> 2.5 km) and near DSG (< 7.5 km). *Plasmodium falciparum* incidence was low and *P. vivax* incidence exhibited a strong persistent increase in 2018. Out of the 82 villages in this zone, 32 (39%) showed Low increasing, 15 (18%) Rainy increasing, five (6%) 2018 increasing and nine (11%) Persistent (*n* = 3, 4%) incidence profiles, geographically distributed throughout the zone.

Another zone was located in the southeastern part of the region, along the border (zone 4). These 24 villages were located near SG landscape and exhibited very low *P. falciparum* incidence and highly seasonal *P. vivax* incidence during the rainy season. The stable overall mean incidence does not reflect the opposing trends of *P. vivax* incidence profiles included in this zone (i.e. Rainy decreasing or Persistent profile). This zone differed from the others in having the highest proportion of *P. vivax* cases in males over the age of 15 years (39.5%), and the lowest proportion of *P. vivax* cases in children (9.8%) (Additional file 1: Fig. S15).

The three remaining zones were only located in the South midland climate, Midland climate and/or were sufficiently far away from at-risk landscapes. They presented low, very low and zero malaria incidence, respectively (Fig. 6).

## Discussion

### Summary of the results

This geo-epidemiological study identified a high diversity of landscapes within the METF region corresponding to a gradient from intact (i.e. dense forest) to heavily human-modified landscapes, characterized by different types of agriculture. Within this diversity of landscapes, three landscapes (DSC, DSG, SG) and a single climate were associated with malaria-affected profiles. Three eco-epidemiological zones with contrasting dynamics were identified by combining these environmental risk factors. One of these zones exhibited longer persistence of *P. falciparum*, up until 2018; another zone showed high *P. vivax* incidence after 2018; and the third displayed stable seasonal *P. vivax* incidence without *P. falciparum*.

### The role of climate was consistent with the literature

One major climate (Midland climate) was linked to malaria-affected profiles irrespective of species or spatial scale. This climate presented optimal conditions for mosquito and parasite development, according to the literature: moderate rainfall between April and October, an annual mean temperature between 18 and 32 °C, and limited temperature variation [27, 28].

### Forest is not a homogeneous environment against malaria

Residing in villages at the forest fringe or inside forested areas was identified as a risk factor for malaria infection in the GMS [29]. Forest covers 76% of the surface of the METF region. Of the 19 landscapes (17 at the region scale and two specifically identified in the Northern Township), 13 landscapes (70%) had ≥50% of their surface covered by forests. An association of sparse and dense forest only was identified as the closest landscape for most villages. This illustrates how in this study area, most of the villages were at the forest fringe or inside the forest. Yet we previously identified heterogeneous malaria transmission within this region [14]. Only three landscapes were associated with malaria-affected incidence profiles, confirming the heterogeneity of forested environments relatively to malaria risk.

### The interface between forest and agricultural lands is at high risk of malaria in the Northern Township

In the Northern Township, DSC and DSG landscapes were associated with malaria-affected incidence profiles. They stood out due to their high proportion of forest (≈90%) fragmented by patches of cropland or grassland/shrubland. Another landscape (Sparse forest, cropland) that was not associated with incidence profile, was highly fragmented but had a higher cover of cropland (32%) and a lower proportion of forest (58%), especially dense forest (20%). The other landscapes had the same proportion of forest cover, but the forest was less fragmented by agricultural patches. In the literature, the role of forest fragmentation in malaria transmission, specifically with respect to the type and proportion of forest or agriculture, remains unclear [30–33]. These findings highlight that, in the Northern Township, a balance between three components (the relative proportions of forest cover, forest fragmentation and agricultural fields) defines at-risk landscapes for malaria, which combine sustained human presence in farms and* Anopheles*-favourable environments in nearby forest [34–36].

In addition, we identified specific dynamics in relationships with specific agricultural types. Long *P. falciparum* persistence and stable *P. vivax* incidence (zone 1) corresponded to cropland located in broad valley bottoms, indicative of inundated rice paddies. Shorter *P. falciparum* persistence and increasing *P. vivax* incidence (zone 3) corresponded to patches of grassland/shrubland located on slopes, a pattern matching traditional Karen *taung yar* agriculture, a rotational farming, 10-year cycle which is practised on slopes [37]. This suggests that the type of agriculture in forested areas could be used as a proxy for topographical and land-use conditions that support heterogeneous dynamics and malaria persistence. It is also possible that inundated rice fields, when located at the forest fringe, directly support higher transmission.

In the Northern Township, the persistent malaria burden in children under the age of 5 years suggests that transmission may still take place within villages, while the lower incidence in adults may be explained by partial immunity.

### The specific context of the southeast border

In the southeast border area of the study region, eco-epidemiological zone 4 stood out due to its proximity to the SG landscape associated with highly seasonal *P. vivax* cases in adult males and few *P. falciparum* cases. Two factors may have contributed to this unique dynamic. First, the predominance of *P. vivax* over *P. falciparum* likely resulted from longer access to early diagnosis and treatment for malaria, which depletes the *P. falciparum* reservoir faster than that of *P. vivax* [2, 12]. Second, a combination of environmental changes and occupational exposure may also explain this specific epidemiology. In contrast with the Northern Township, the landscape associated with this dynamic has been mostly covered by sparse forest and agricultural land since intense deforestation between 2004 and 2010 [26]. The exact occupation responsible for higher malaria exposure is unclear, but we hypothesize that it may be related to seasonal farming activities carried out outside villages. Such activities usually involve adult males (e.g. seasonal migrant workers), who live on the farm over prolonged periods from the onset of the rainy season until harvest. The vectors to which they are potentially exposed may originate from small patches of sparse vegetation (e.g. along streams) rather than rarer, dense forest or forest fringes.

### Study strengths

Using the unique dataset of weekly malaria case reports collected by the METF MP network, we were able to study the association between malaria incidence and forested environments in unprecedented detail. This dataset allowed the study of malaria incidence at village scale without requiring further spatial aggregation. Compared to the annual parasite index, incidence profiles allowed the temporal characterization of intra- and inter-annual variation in amplitude, seasonality, and tendency over a 4-year period. They enabled us to identify villages that were long-term hotspots or showed malaria persistence. *Plasmodium falciparum* and *P. vivax* dynamics could also be studied in parallel or combined. This was especially relevant as METF interventions were targeted at *P. falciparum* and had less impact on *P. vivax*, leading to different persistence patterns.

In addition, malaria transmission through mosquito bites can occur within villages or in remote locations where there is human activity and the precise location of infection is usually unknown. It is therefore difficult to estimate the distance between households and transmission sites, as this depends on the particular activity carried out (e.g. agriculture, logging, etc.), geographical accessibility (topography, road access) and means of travel. To overcome this, we described the climate and landscape over the entire region regardless of the location of the villages. We also relied on high-resolution land use and land cover data, and used fragmentation indices, which are key determinants of landscapes at risk for malaria in the Northern Township. Fragmentation indices are important for the characterization of interfaces between types of natural environment, such as forest, and human-modified environment (e.g. cropland), and where the probability of contact between vectors and humans is higher.

To study the association between malaria incidence profiles and environment, we chose to use a tree-based model and random forests to overcome intrinsic issues associated with regression models, i.e. small sample size for some incidence profiles, unknown distribution, and potential correlation or interaction between covariates (i.e. climate and landscape).

This analysis ultimately identified eco-epidemiological zones, which included villages within a similar risk area but with heterogeneous malaria incidence profiles. Beyond following World Health Organization recommendations concerning malaria risk stratification (i.e. based on transmission and receptivity), this approach is of interest in an elimination context where outbreaks are more stochastic, as illustrated by *P. falciparum* incidence profiles [9]. These results could help in the targetting and planning of surveillance by identifying villages where outbreaks are more likely. They could also be used for the planning of routine activities to target additional effort where it is needed.

### Study limitations

The first challenge in this study was the overall low malaria incidence in the METF region over the study period. Indeed, 81% of the villages were classified as having a very low incidence profile for *P. falciparum*, and 69% a very low incidence profile for *P. vivax*. The other 10 profiles only corresponded to a small number of villages (from one to 46). The imbalance in these distributions limited the subsequent analysis of factors associated with each incidence profile and the use of regression models. The CIT analysis mostly distinguished malaria-affected from malaria-free incidence profiles, except for *P. vivax* incidence in the Northern Township. However, by combining environmental factors associated with both *Plasmodium* species, we were able to describe sub-regional eco-epidemiological zones, which corresponded to specific local environments and were characterized by specific trends and at-risk populations. These results suggested a relationship between malaria dynamics and environment at a spatial scale higher than that of the village. These findings agree with previous reports of *P. falciparum* prevalence hotspots clustering within a radius of 10 km in this region [12].

We relied on the incidence of clinical cases of malaria diagnosed by MP as a proxy of malaria transmission. This assumption likely led to the underestimation of transmission, since individuals with sufficient immunity will not necessarily develop clinical malaria episode upon infection. On the other hand, using the incidence of clinical cases of malaria to determine transmission, we could not distinguish *P. vivax* infections resulting from an infective bite from those due to a relapse. This could have resulted in an overestimation of *P. vivax* transmission, or inability to identify some seasonal patterns (e.g. zone 1). However, in the analysis of 1441 recurrent episodes pooled from two trials conducted in the same setting, 95% of *P. vivax* recurrences (88% relapses and 12% reinfections) were detected upon active follow-up, while only 5% were associated with sufficient symptoms to trigger an intercurrent consultation [38]. In these trials, active participant follow-up may have led to early detection and preventive treatment of some relapses before they became clinical. Despite this potential underestimation, clinical relapses are thought to only moderately modify the incidence dynamics.

Finally, even if the LULC classification allowed a 10-m resolution, validated with field observations, there were still some classes that were difficult to distinguish, i.e. roads from rivers, and agricultural patches from low vegetation. These elements of the environment may modulate vector presence and thus the association of environmental factors with incidence profiles. In addition, this study focused on land cover patterns, such as fragmentation and vegetation density. Identifying differences in floristic composition across forested areas was not feasible here, but could allow for further characterization of forest heterogeneity.

### Generalizations and perspectives

The aims of this study of malaria dynamics at the local level were to improve our understanding of the epidemiology of this disease in an increasingly heterogeneous context and to contribute to intervention planning. The analysis identified eco-epidemiological zones with higher malaria incidence where specific interventions could be targeted (for more details, see [14]). These zones aggregated villages sharing similar environments, and thus receptivity, beyond local incidence heterogeneities. The use of this type of zoning is valuable for the identification of areas prone to malaria resurgence, where surveillance is strategic. Indeed, at low transmission levels, favourable environmental conditions are necessary, but not sufficient on their own, as the presence of a parasite reservoir is also required.

Health systems often rely on the collection of aggregated surveillance data (geographically, temporally, and by age). This study demonstrates how rich surveillance data could inform an understanding of malaria epidemiology and intervention planning, especially in a highly heterogeneous context. Our results support the current trend of investing in state-of-the-art epidemiological information systems. However, the size of the eco-epidemiological zones reported here suggest that analyses performed at the scale of a health facility catchment area (e.g. a dozen villages) could yield relevant results for larger regions or country-wide studies.

Additionally, eco-epidemiological zones were characterized by differences in climate, landscape, and agricultural systems, which are biologically relevant factors that may be applicable to other forested areas. Studying structural environmental factors at a broader scale in the GMS could help identify areas favourable to *Anopheles* presence and determine proxies of human exposure. Combining existing lower resolution LULC with other remotely sensed data sources (forest loss data, or the detection of logging or fires leading to deforestation) could provide community-level assessments of receptivity and human activities in or at the fringe of forested areas [39].

## Conclusions

This study shows that the generic term ‘forest’ hides a substantial diversity of environments, which can differ with respect to malaria persistence, dynamics and populations at risk. This diversity highlights the fact that there is no one-size-fits-all approach for forest malaria with respect to policy planning, modelling approaches and implementation. The existence of diverse human populations within heterogeneous eco-epidemiological settings should be taken into consideration when designing malaria elimination strategies.

### Supplementary Information

**Additional file 1: Figure S1.** Maps showing incidence profiles. Mapped villages in the malaria elimination task force (METF) target region according to malaria incidence profiles. **A*** Plasmodium falciparum* incidence profiles and **B*** Plasmodium vivax* incidence profiles; inset shows a map of the Hpapun/Mutraw administrative township (Northern Township). This original figure presents raw data and results published previously [14].** Method S1**. Landscape. **Figure S2.** Villages receiving mass drug administration (MDA) according to the year. MDAs were conducted in high prevalence hotspots identified by prevalence surveys [13]. At the beginning of the METF program there was little knowledge about the location of malaria hotspots. Prevalence surveys were therefore conducted in randomly selected villages, and followed an east (closer to Thailand) to west pattern related to the gradual deployment of the program from the border with Thailand towards the interior of Myanmar. The spatial analysis of prevalence measured in the first surveys revealed spatial clustering of hotspot villages: there was a higher likelihood of finding a hotspot within 10 km of another one [12]. In addition, hotspots also displayed a higher incidence of clinical malaria. After 2015, surveys were targeted based on these two criteria. As a result of this, MDAs were concentrated in the Northern Township. **Figure S3.** Climate description. Ombrothermic diagram of the five climates identified at region scale. Temperature data of June, July and August were excluded from the analysis because of too many missing data points due to cloud cover in the rainy season (dotted lines). Rainfall data of November, December, January, February and March, corresponding to the cold dry season, were also excluded because of the occurrence of rare, local and intense thunderstorms. **Figure S4.** Land use and land cover classification description.** A** Map of the land use and land cover (LULC) classification.** B** Distribution of the LULC class at the region scale and the Northern township scale. **Figure S5.** Landscape map at region scale.** A** Landscape map.** B** Altitude map. **Figure S6.** Landscape description at region scale. LULC description of landscapes representing more than 1% of the region surface, included in association analysis (**A**) or excluded (**B**).** C** Description of landscapes identified at region scale according to altitude. **Figure S7.** Covariates selection. Landscapes were selected according to their median distance to villages at region (**A**) and township (**B**) scale. Landscapes with a median ≤ 10 km (red dotted and solid lines) were included. Sensitive analysis was conducted with cut-offs of 5 km (grey dotted line) and 15 km (blue dotted and solid lines). Landscapes selected with the 10-km and 5-km cut-offs were similar. **Figure S8.** Landscapes in the Northern Township.** A** Description of the two landscapes specifically identified in the Northern Township according to the LULC classification. The other landscapes were also identified at region scale and described in figure S5.** B** Map of the most common landscapes identified at the township scale. **Figure S9.** Study association between malaria incidence profiles and environment in the METF region by conditional random forests (CRF). A 10-km cut-off was applied to selected landscape covariates.** A** CRF of* Plasmodium falciparum* incidence profiles depending on landscapes, climates, and MDA before March 2016.** B** CRF of* Plasmodium vivax* incidence profiles depending on landscapes, climates, and MDA after February 2016. **Figure S10.** Study association between malaria incidence profiles and environment in the Northern Township by CRF. A 10-km cut-off was applied to select landscape covariates.** A** CRF of* P. falciparum* incidence profiles depending on landscapes, climates, and MDA before March 2016.** B** CRF of* P. vivax* incidence profiles depending on landscapes, climates, and MDA after February 2016. **Figure S11.** Association study between malaria incidence profiles and environment in METF region by conditional inference trees (CIT). A 15-km cut-off was applied to select landscape covariates.** A** CIT of* P. falciparum* incidence profiles depending on landscapes, climates, and MDA before March 2016.** B** Location of villages depending on their nodes obtained with* P. falciparum* CIT.** C** CIT of* P. vivax* incidence profiles depending on landscapes, climates, and MDA after February 2016.** D** Location of villages depending on their nodes obtained with* P. vivax* CIT. In CIT, ‘node’ refers to a group of villages. **Figure S12.** Association study between malaria incidence profiles and environment in METF region by CRF. A 15-km cut-off was applied to select landscape covariates.** A** CRF of* P. falciparum* incidence profiles depending on landscapes, climates, and MDA before March 2016.** B** CRF of* P. vivax* incidence profiles depending on landscapes, climates, and MDA after February 2016. **Figure S13.** Association study between malaria incidence profiles and environment in the Northern Township by CIT. A 15-km cut-off was applied to select landscape covariates.** A** CIT of* P. falciparum* incidence profiles depending on landscapes, climates, and MDA before March 2016.** B** Location of villages depending on their nodes obtained with P. falciparum CIT.** C** CIT of* P. vivax* incidence profiles depending on landscapes, climates, and MDA after February 2016.** D** Location of villages depending on their nodes obtained with* P. vivax* CIT. In CIT, ‘node’ refers to a group of villages. **Figure S14.** Association study between malaria incidence profiles and environment in the Northern Township by CRF. A 15-km cut-off was applied to select landscape covariates.** A** CRF of* P. falciparum* incidence profiles depending on landscapes, climates, and MDA before March 2016.** B** CRF of* P. vivax* incidence profiles depending on landscapes, climates, and MDA after February 2016. **Figure S15.** Description of eco-epidemiological zones according to age and gender malaria cases. Proportion of reported* P. falciparum* (**A**) and* P. vivax* (**B**) cases during the study period by malaria posts depending on gender (male, female), age, and eco-epidemiological zones. The total number of malaria cases is specified in the legend. Five classes described cases by age and gender: woman and men between 0 and 5 years (W/M 0-5), women between 5 and 15 years (W 5-15), women older than 15 years (W 15-99), men between 5 and 15 years (M 5-15), men older than 15 years (M 15-99). **Table S1.** Eco-epidemiological zone construction. Tables present the number of villages in each node and proportions in brackets.** A*** Plasmodium falciparum* zone construction by cross analysis between CIT results at region and township scale.** B*** Plasmodium vivax* zone construction by cross analysis between CIT results at region and township scale.** C** Cross analysis between* P. falciparum* and* P. vivax*.* DSC* Dense forest, sparse forest, cropland landscape;* DSG* dense forest, sparse forest, grassland landscape;* SG* sparse forest, grassland landscape;* M* midland climate;* SM* south midland climate;* N* node.

## Data Availability

The data analysed for this study are available upon request to the Mahidol Oxford Tropical Medicine Research Unit data access committee: https://www.tropmedres.ac/units/moru-bangkok/bioethics-engagement/data-sharing.

## Abbreviations

CIT: Conditional inference trees
CRF: Conditional random forests
DSC: Dense forest, sparse forest, cropland landscape
DSG: Dense forest, sparse forest, grassland landscape
GMS: Greater Mekong subregion
HAC: Hierarchical ascendant clustering
LULC: Land use land cover
MDA: Mass drug administration
METF: Malaria elimination task force
MP: Malaria post
PCA: Principal components analysis
SG: Sparse forest, grassland landscape
