# Supplementary material for: ‘Forest malaria’ in Myanmar? Tracking transmission landscapes in a diversity of environments
Source: Parasit Vectors. 2023 Sep 12;16:324. doi: 10.1186/s13071-023-05915-w (PMC10498628; doi:10.1186/s13071-023-05915-w)
Supplement: Supplementary file 1 — Additional file 1: Figure S1. Maps showing incidence profiles. Mapped villages in the malaria elimination task force (METF) target region according to malaria incidence profiles. A Plasmodium falciparum incidence profiles and B Plasmodium vivax incidence profiles; inset shows a map of the Hpapun/Mutraw administrative township (Northern Township). This original figure presents raw data and results published previously [14]. Method S1. Landscape. Figure S2. Villages receiving mass drug administration (MDA) according to the year. MDAs were conducted in high prevalence hotspots identified by prevalence surveys [13]. At the beginning of the METF program there was little knowledge about the location of malaria hotspots. Prevalence surveys were therefore conducted in randomly selected villages, and followed an east (closer to Thailand) to west pattern related to the gradual deployment of the program from the border with Thailand towards the interior of Myanmar. The spatial analysis of prevalence measured in the first surveys revealed spatial clustering of hotspot villages: there was a higher likelihood of finding a hotspot within 10 km of another one [12]. In addition, hotspots also displayed a higher incidence of clinical malaria. After 2015, surveys were targeted based on these two criteria. As a result of this, MDAs were concentrated in the Northern Township. Figure S3. Climate description. Ombrothermic diagram of the five climates identified at region scale. Temperature data of June, July and August were excluded from the analysis because of too many missing data points due to cloud cover in the rainy season (dotted lines). Rainfall data of November, December, January, February and March, corresponding to the cold dry season, were also excluded because of the occurrence of rare, local and intense thunderstorms. Figure S4. Land use and land cover classification description. A Map of the land use and land cover (LULC) classification. B Distribution of the LULC class at t [file 13071_2023_5915_MOESM1_ESM.docx]

# Additional file

## Figure S1. Incidence profiles map. Maps of villages in the METF target region according to malaria incidence profiles. A) *Plasmodium falciparum* incidence profiles and (B) *Plasmodium vivax* incidence profiles with a zoom on the Northern Township. This original figure presents data and results published previously [14].


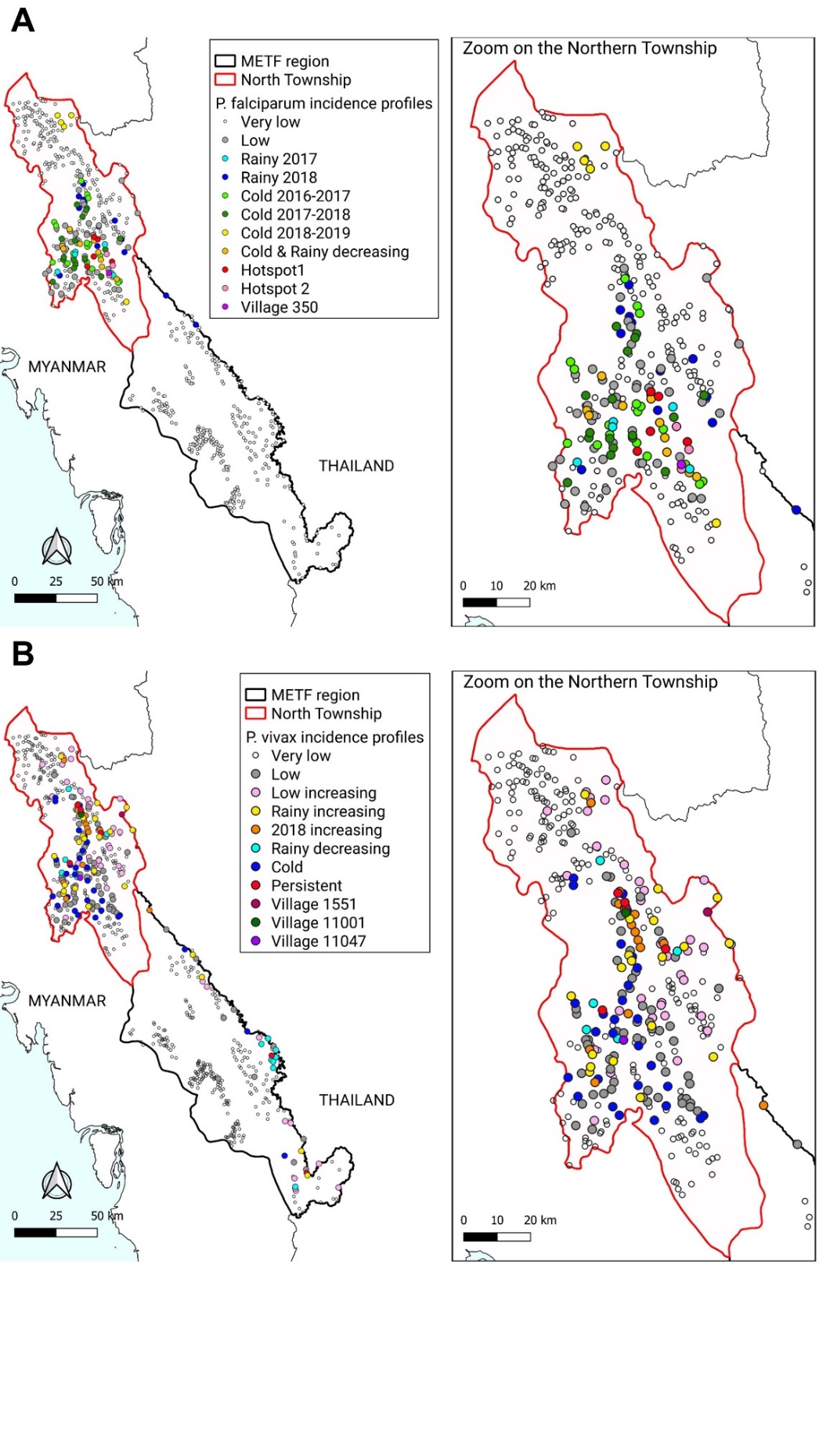


#### Method S1. Landscape.

To characterize landscape diversity in detail, a variable summing up land use land cover (LULC), altitude and slope data and fragmentation indices were constructed.

The LULC classification of high resolution was composed of 10 classes: built-up, water, road, wetland, bare soil, grass/shrubland, crop field, dense plantation, sparse forest and dense forest. The road class was not considered in this analysis because of missing information, especially mountains path. Water and wetland classes were merged in one class.

Slope and altitude summary were estimated for each hexagon: mean, median, minimum, maximum, standard deviation, and percentage of 0-5° slope, 5-10° slope, >10° slope^[[1]](#footnote-1)^.

For each hexagon, fragmentation indices based on LULC classification were also estimated. Fragmentation indices were: percentage cover area for each class of LULC, number of class, number of patch (overall, for each class), mean patch size (overall, for each class), median patch size, (overall, for each class), greatest patch area (overall, for each class), largest patch index (overall, for each class), overall patch density, perimeter area ratio for forest classes^^[[2]](#footnote-2)^^. A patch corresponds to a homogeneous surface composed of only one LULC class and surrounded by different LULC classes. Patches less than 100m² were excluded from the analysis because they were under satellite spatial resolution.

Landscape variable was constructed by an hierarchical ascendant clustering (HAC) on principal components. Hexagons with LULC missing information greater than or equal to 5% of their surfaces were not included in the classification. These hexagons corresponded to the border of the Malaria Elimination Task Force (METF) zones. This selection was made to maintain comparability of fragmentations indices between hexagons. Principal components explaining 99% of variance were selected in the HAC, assuming residual information as noise.

The distance to each landscape was calculated for each village using a buffer zone of 500m around villages to take into account malaria post groupings. A logarithm transformation was applied to the distance to consider only close environment by weighting landscapes within 1km around villages.

## Figure S2. Villages receiving a mass drug administration (MDA) according to the year. MDAs were conducted in high prevalence hotspots identified by prevalence surveys [13]. At the beginning of the Malaria Elimination Task Force (METF) program, there was little knowledge about the location of malaria hotspots. Prevalence surveys were therefore conducted in randomly selected villages, and followed a east (closer to Thailand) to west pattern related to the gradual deployment of the program from the border with Thailand towards inside Myanmar. Subsequently, the spatial analysis of prevalence measured in the first surveys revealed spatial clustering of hotspot villages: there was a higher likelihood of finding a hotspot within 10km from another one [12]. In addition, hotspots also displayed higher incidence of clinical malaria. After 2015, surveys were targeted based on these two criteria. As a result, MDAs concentrated in the Northern Township.


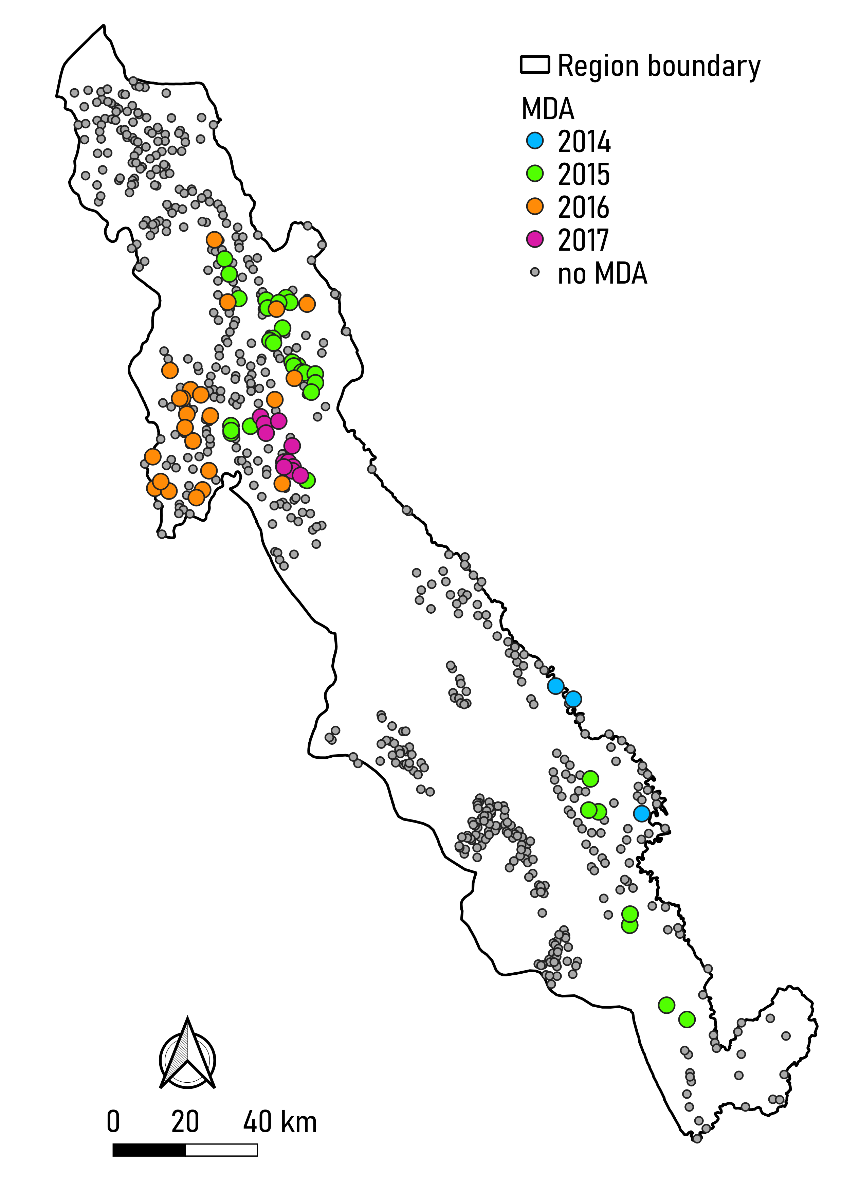


## Figure S3. Climate description. Ombrothermic diagram of the five climates identified at region scale. Temperature information of June, July and August were excluded from the analysis because of too much missing data caused by clouds due to rainy season (dotted lines). Rainfall information of November, December, January, February and March, corresponding to cold dry season, were also excluded because of the occurrence of rare, locally and intense thunderstorms.


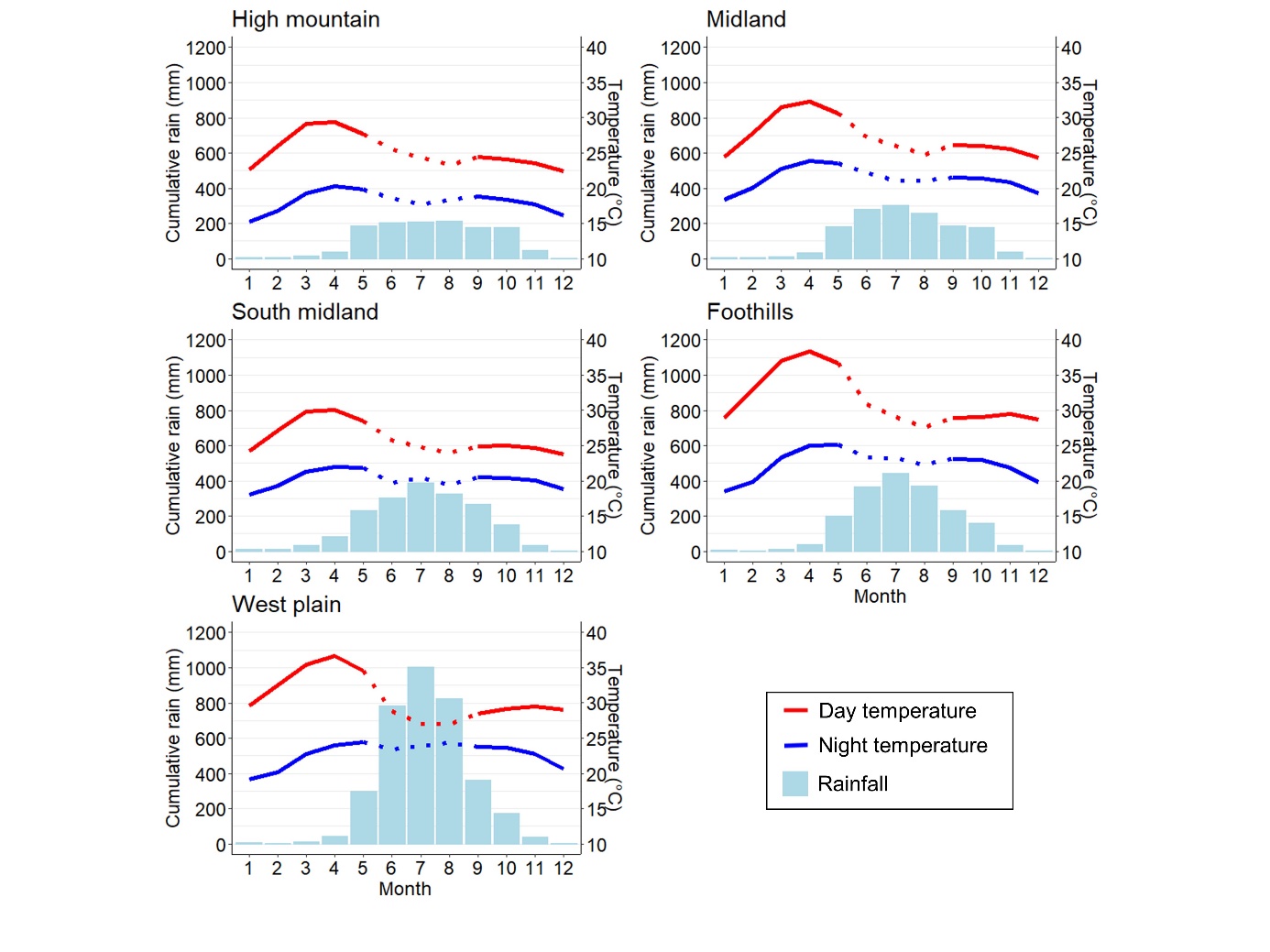


## Figure S4. Land use and land cover classification description. A) Map of the land use and land cover (LULC) classification. B) Distribution of the LULC class at the region scale and the Northern township scale.


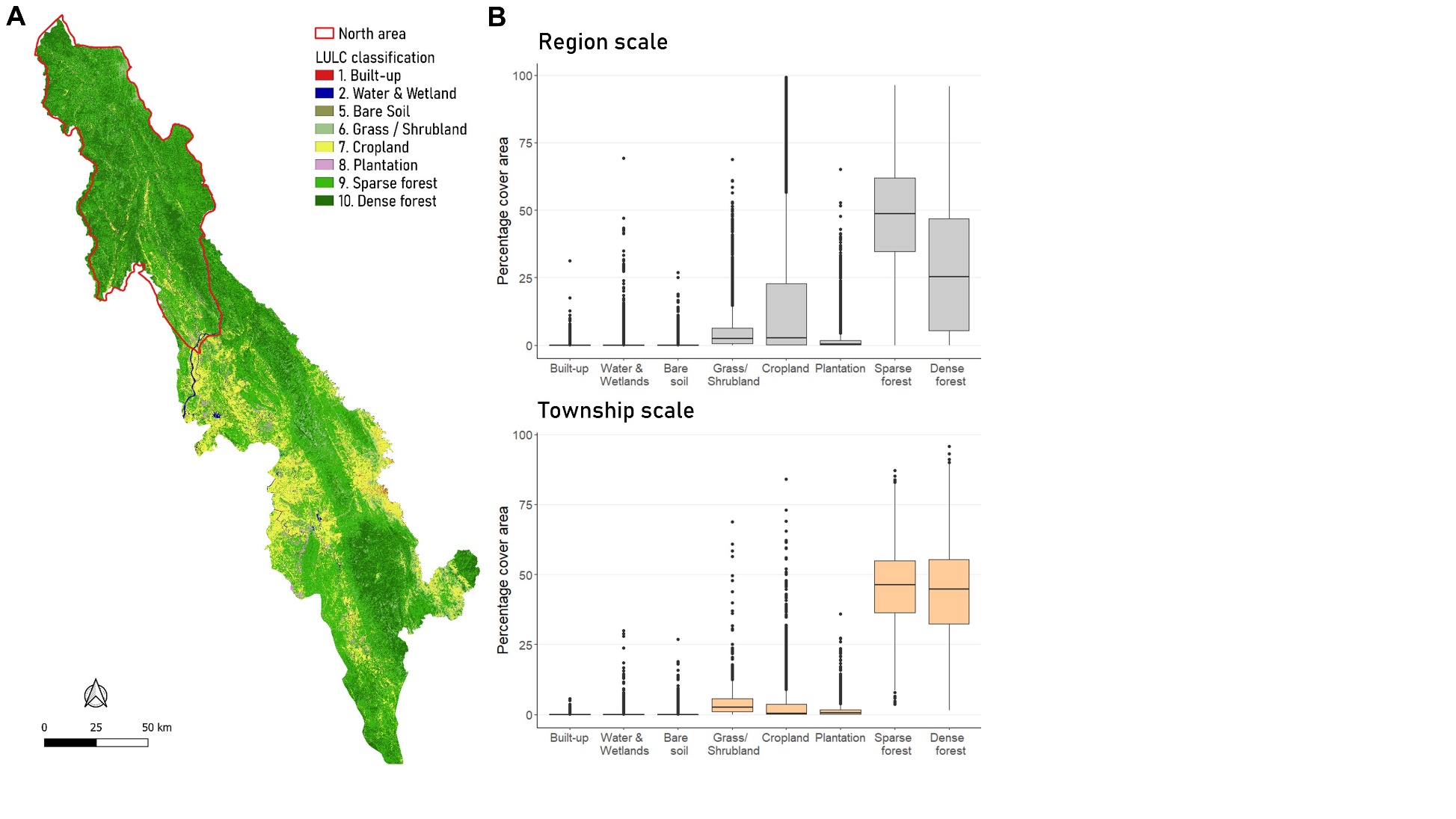


## Figure S5. Landscape map at region scale. A) Landscape map. B) Altitude map.


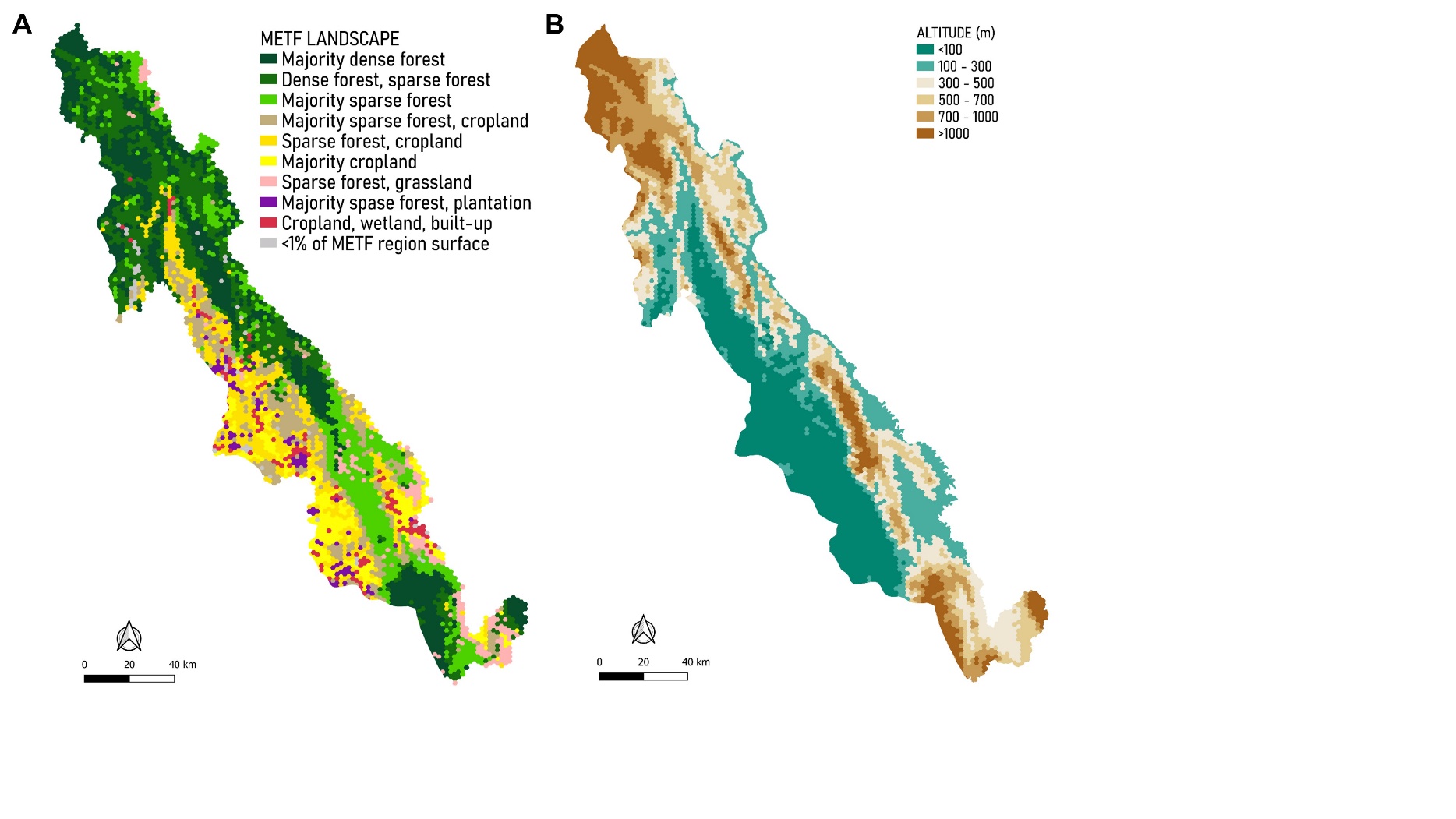


## Figure S6. Landscape description at region scale. Land use and land cover (LULC) description of landscapes representing more than 1% of the region surface, included in association analysis (A) and excluded (B). C) Description of landscapes identified at region scale according to altitude.


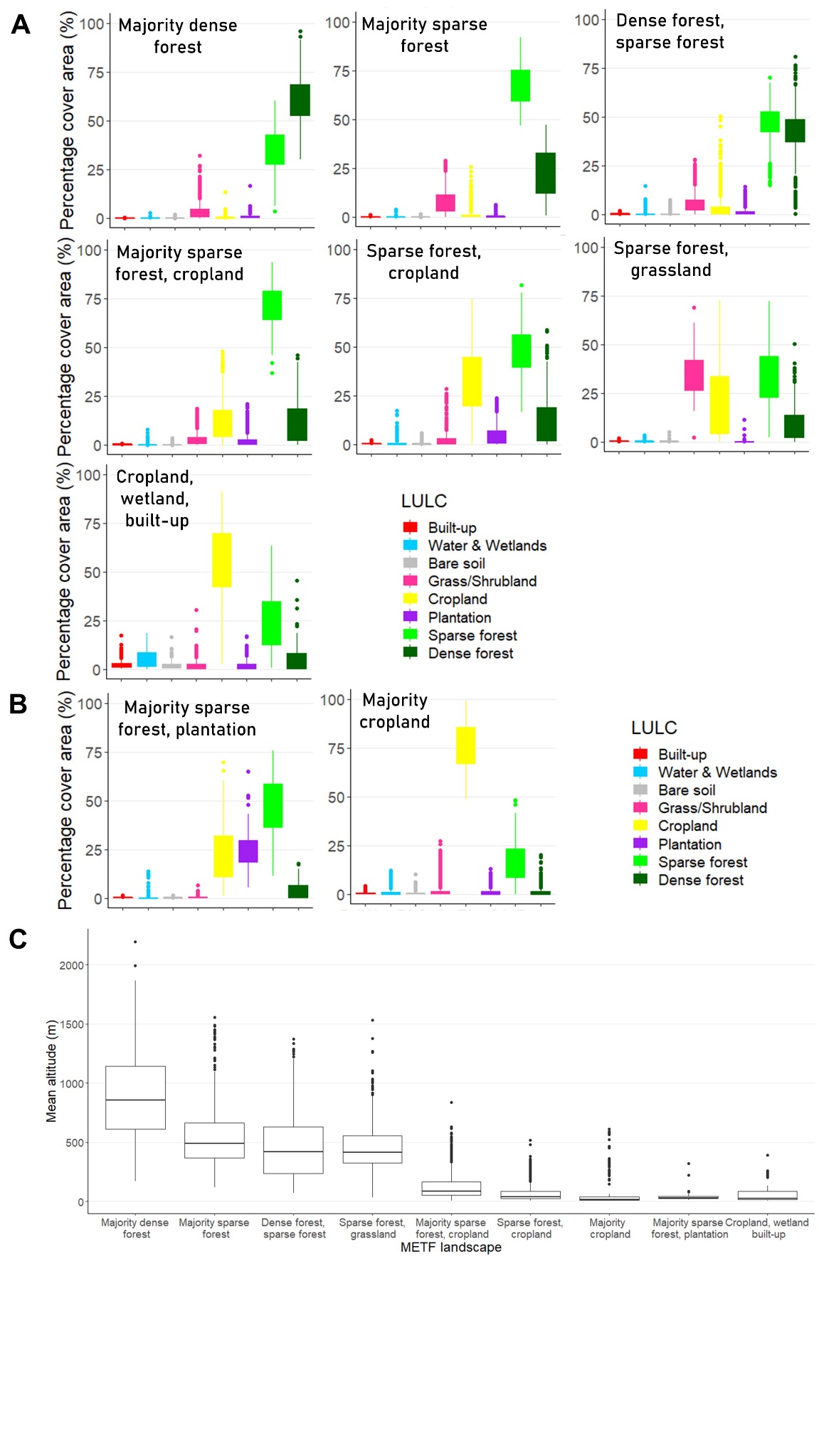


## Figure S7. Covariates selection. Landscapes were selected according to their median distance to villages at region (A) and township (B) scale. Landscapes with a median ≤10km (red dotted and solid lines) were included. Sensitive analysis was conducted with cut-offs of 5km (grey dotted line) and 15km (blue dotted and solid lines). Landscapes selected with the 10km and 5km cut-offs were similar.


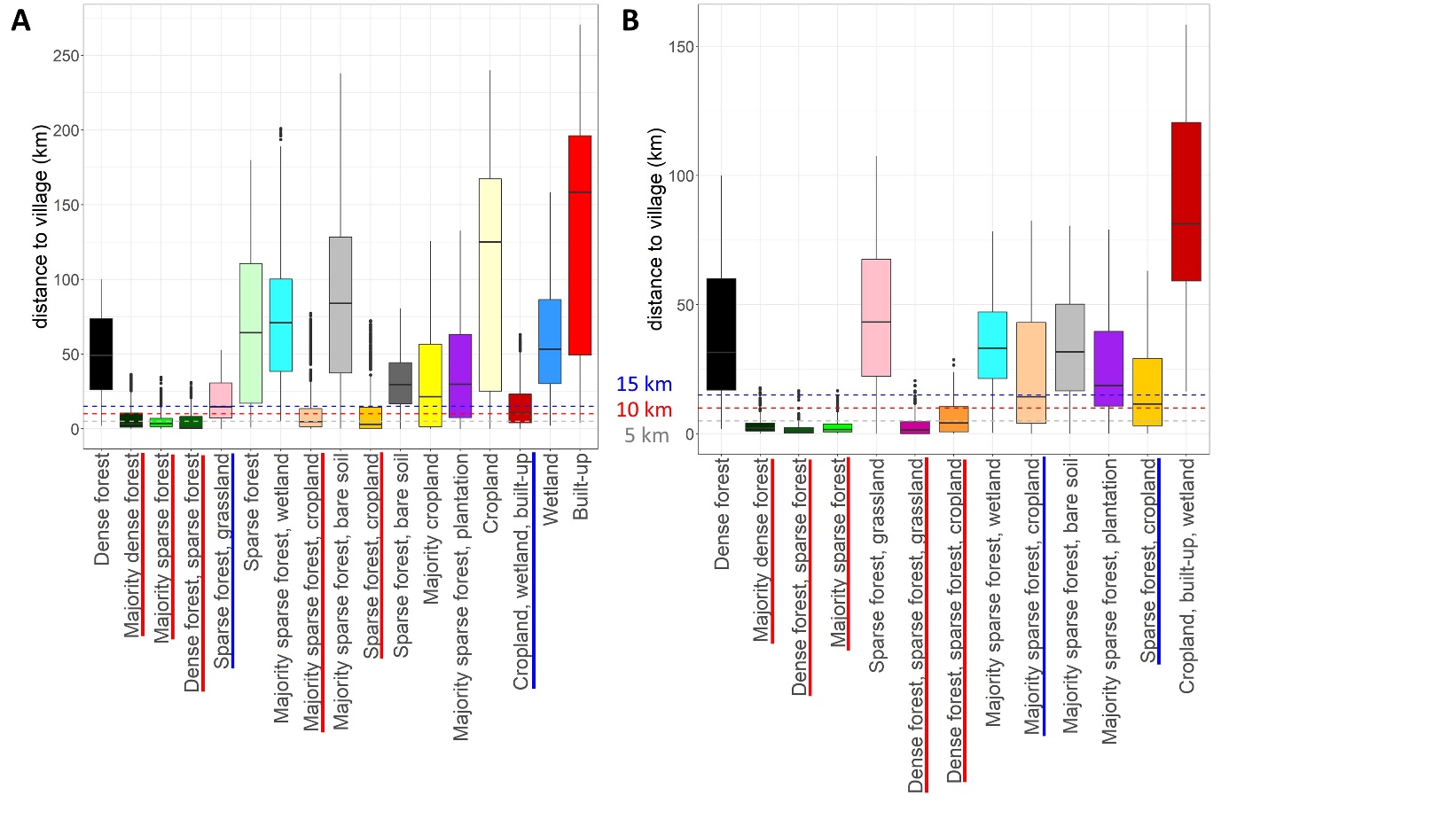


## Figure S8. Landscapes in the Northern Township. A) Description of the two landscapes specifically identified in the Northern Township according to the land use land cover (LULC) classification. The other landscapes were also identified at region scale and described in figure S5. B) Map of the most common landscapes identified at the township scale.

**
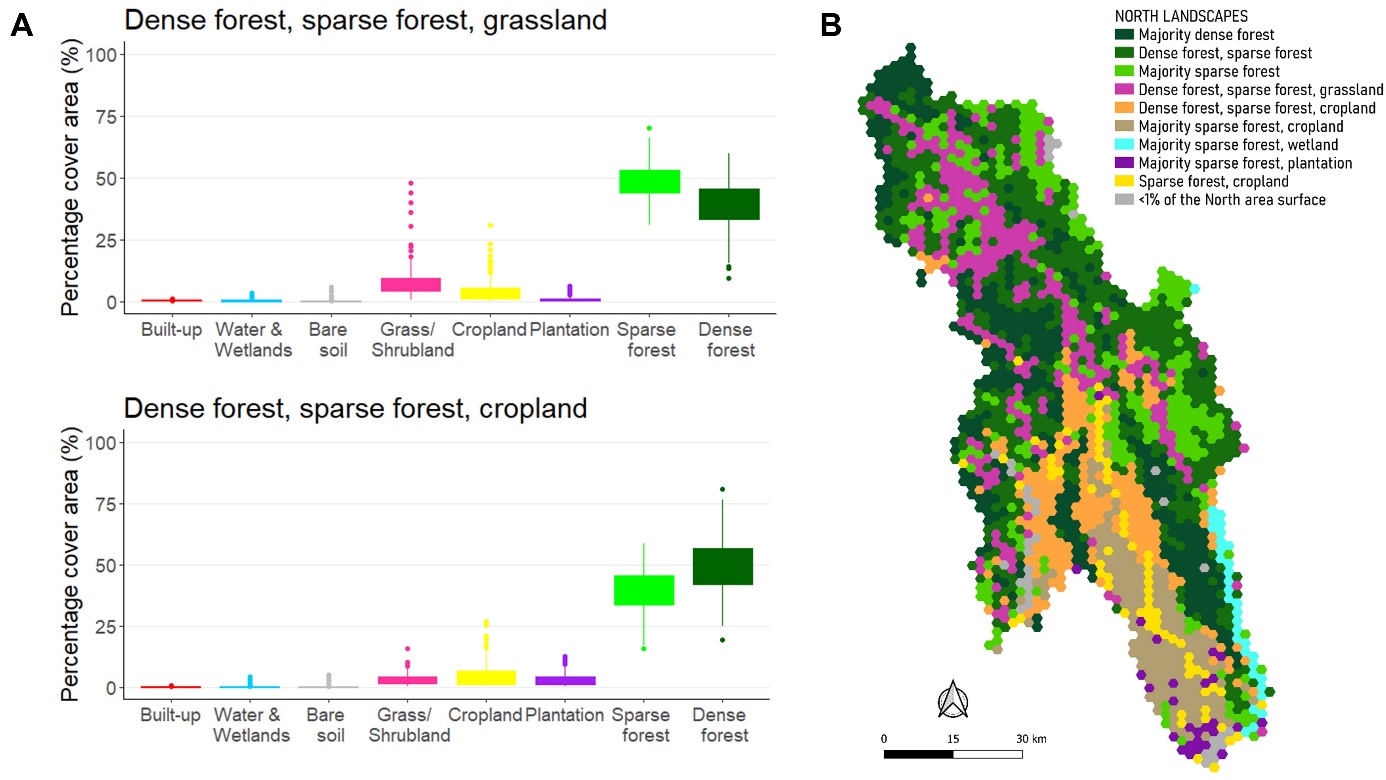
**

## Figure S9. Study association between malaria incidence profiles and environment in METF region by conditional random forest (CRF). A 10 km cut-off was applied to select landscape covariates. A) CRF of *P. falciparum* incidence profiles depending on landscapes, climates, and mass drug administration (MDA) before March 2016. B) CRF of *P. vivax* incidence profiles depending on landscapes, climates, and MDA after February 2016.


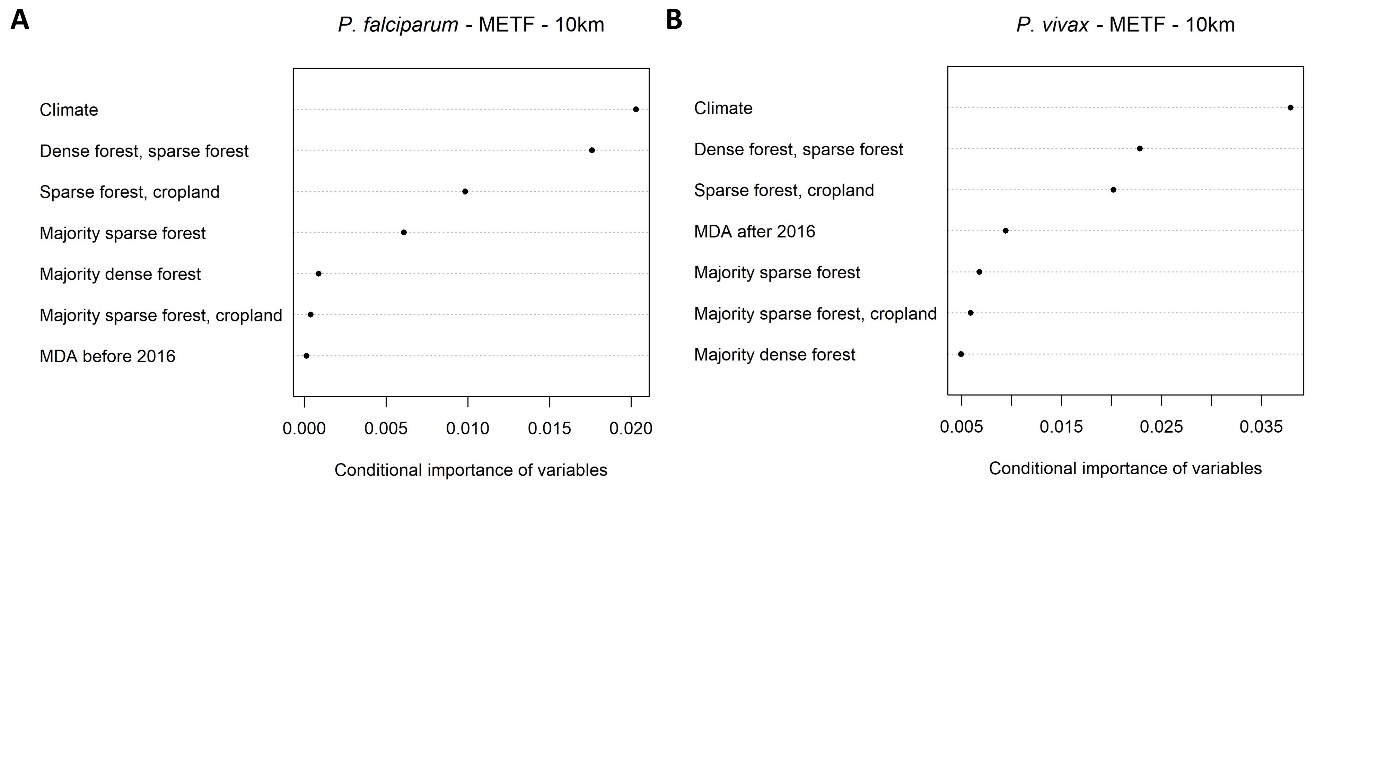


## Figure S10. Study association between malaria incidence profiles and environment in the Northern Township by conditional random forest (CRF). A 10km cut-off was applied to select landscape covariates. A) CRF of *P. falciparum* incidence profiles depending on landscapes, climates, and mass drug administration (MDA) before March 2016. B) CRF of *P. vivax* incidence profiles depending on landscapes, climates, and MDA after February 2016.


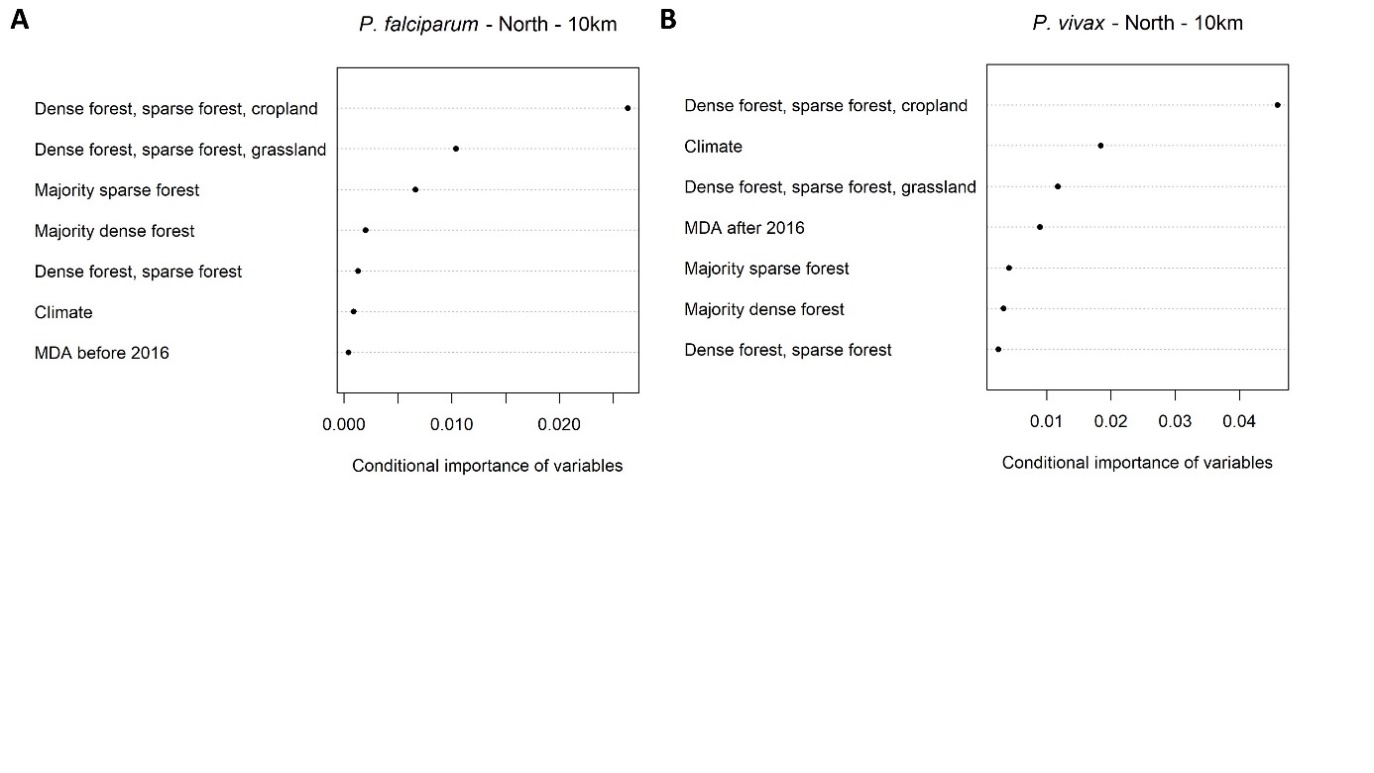


Figure S11. Association study between malaria incidence profiles and environment in METF region by conditional inference trees (CIT). A 15km cut-off was applied to select landscape covariates. **A)** CIT of *P. falciparum* incidence profiles depending on landscapes, climates, and mass drug administration (MDA) before March 2016. **B)** Location of villages depending on their nodes obtained with *P. falciparum* CIT. **C)** CIT of *P. vivax* incidence profiles depending on landscapes, climates, and MDA after February 2016. **D)** Location of villages depending on their nodes obtained with *P. vivax* CIT. In CIT, “node” refers to a group of villages.


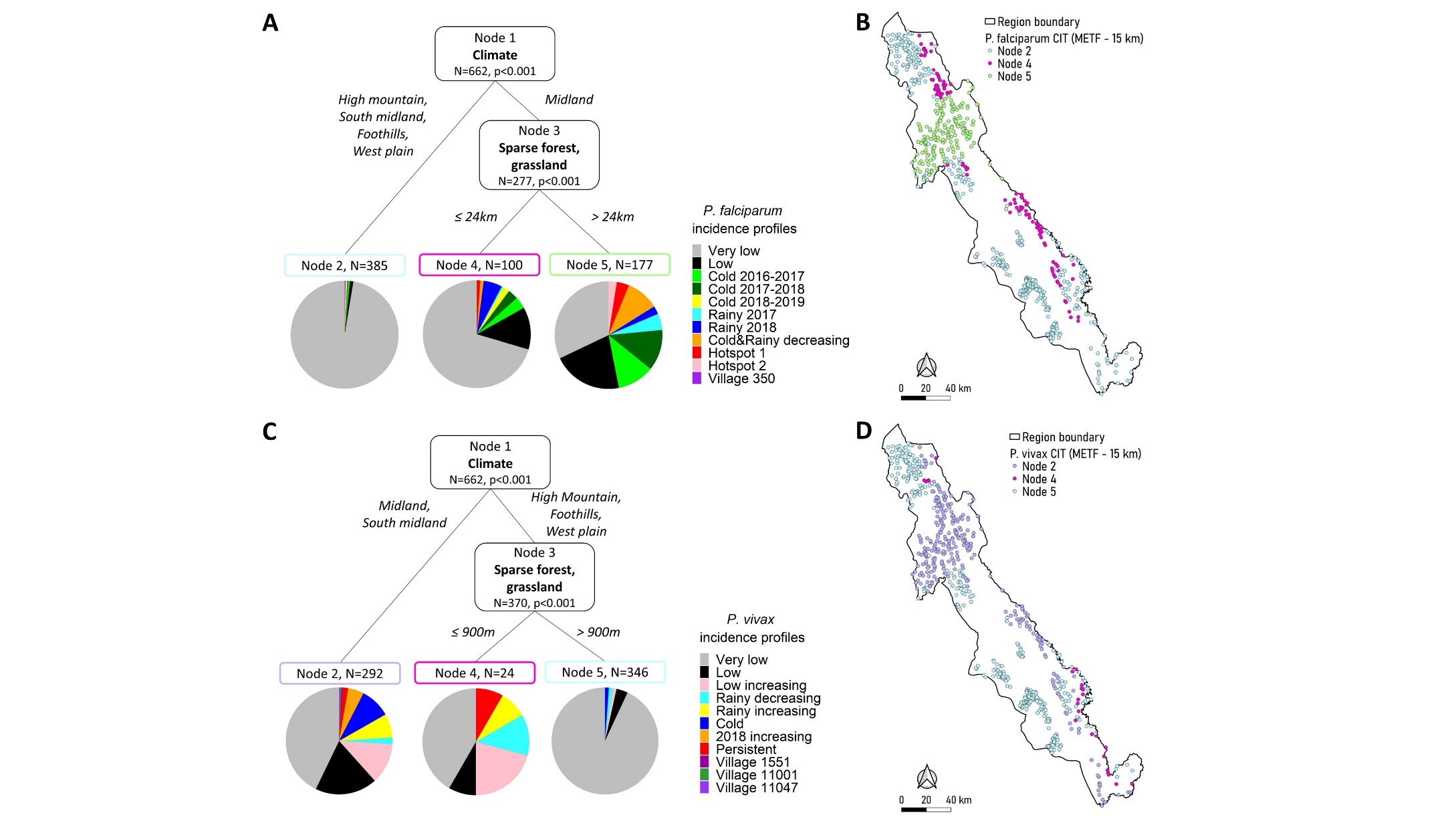


## Figure S12. Association study between malaria incidence profiles and environment in METF region by conditional random forest (CRF). A 15km cut-off was applied to select landscape covariates. A) CRF of *P. falciparum* incidence profiles depending on landscapes, climates, and mass drug administration (MDA) before March 2016. B) CRF of *P. vivax* incidence profiles depending on landscapes, climates, and MDA after February 2016.


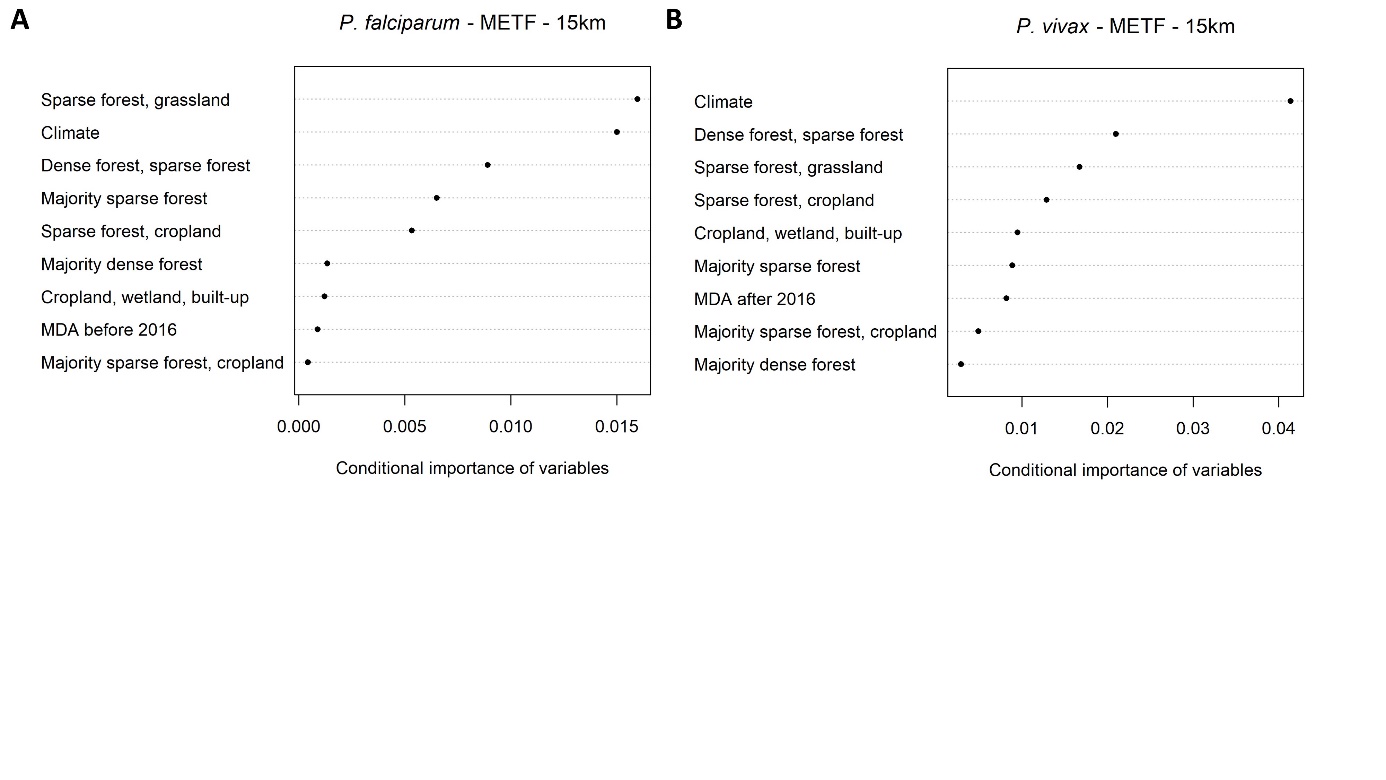


Figure S13. Association study between malaria incidence profiles and environment in the Northern Township by conditional inference trees (CIT). A 15km cut-off was applied to select landscape covariates. **A)** CIT of *P. falciparum* incidence profiles depending on landscapes, climates, and mass drug administration (MDA) before March 2016. **B)** Location of villages depending on their nodes obtained with *P. falciparum* CIT. **C)** CIT of *P. vivax* incidence profiles depending on landscapes, climates, and MDA after February 2016. **D)** Location of villages depending on their nodes obtained with *P. vivax* CIT. In CIT, “node” refers to a group of villages.


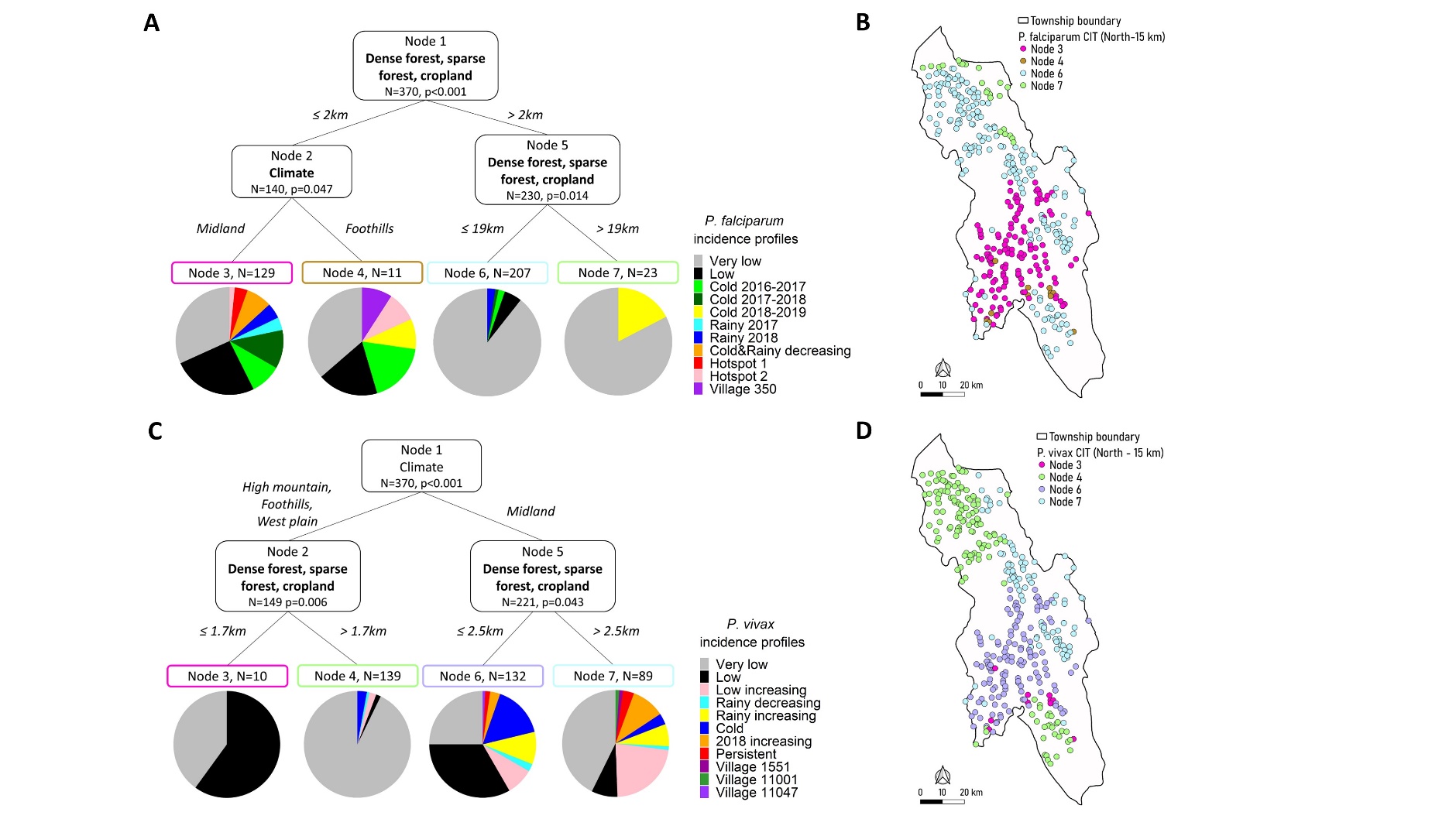


## Figure S14. Association study between malaria incidence profiles and environment in the Northern Township by conditional random forest (CRF). A 15km cut-off was applied to select landscape covariates. A) CRF of *P. falciparum* incidence profiles depending on landscapes, climates, and mass drug administration (MDA) before March 2016. B) CRF of *P. vivax* incidence profiles depending on landscapes, climates, and MDA after February 2016.


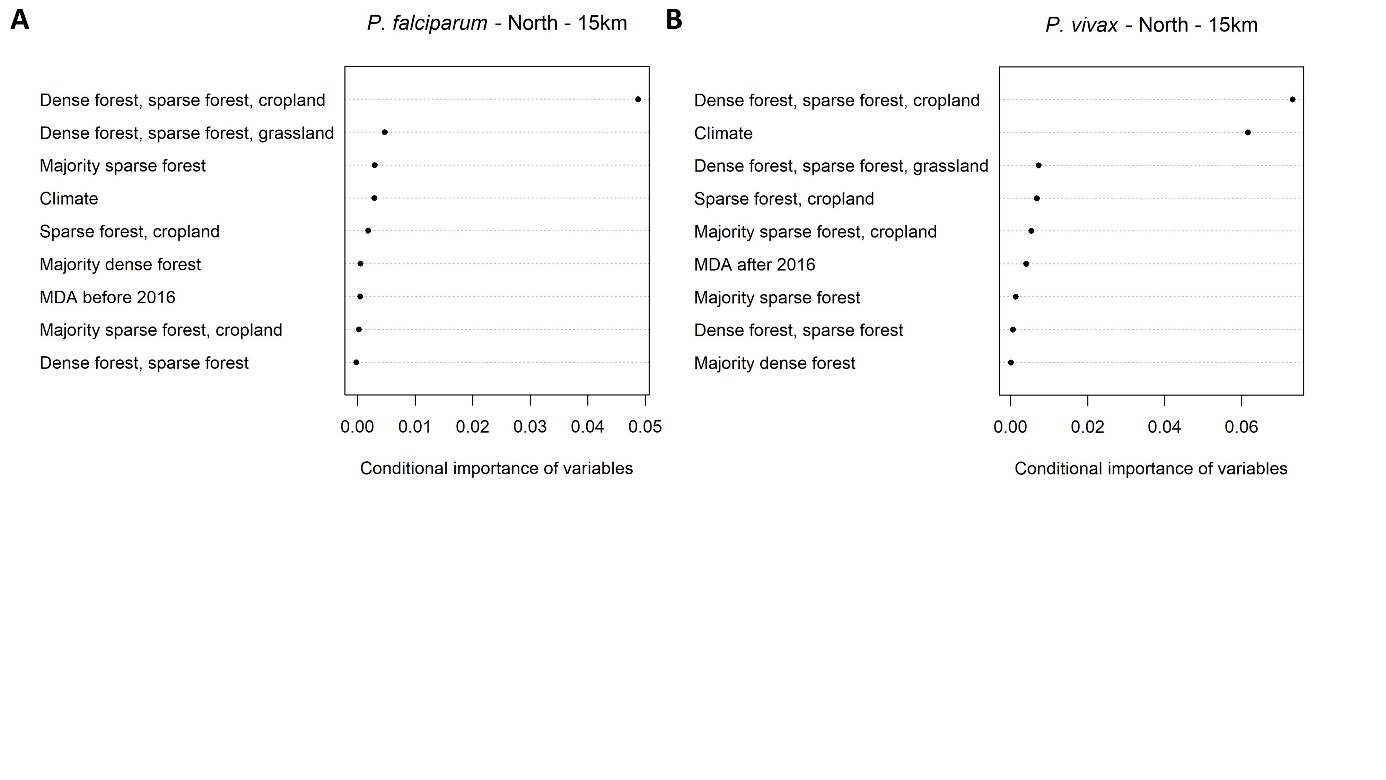


## Figure S15. Description of eco-epidemiological zones according to age and gender malaria cases. Proportion of reported *P. falciparum* (A) and *P. vivax* (B) cases during the study period by malaria posts depending on gender (male, female), age, and eco-epidemiological zones. The total number of malaria cases is specified in the abscise legend. Five class described age and gender cases: woman and men between 0-5 years (W/M 0-5), woman between 5-15 years (W 5-15), woman older than 15 years (W 15-99), men between 5-15 years (M 5-15), men older than 15 years (M 15-99).


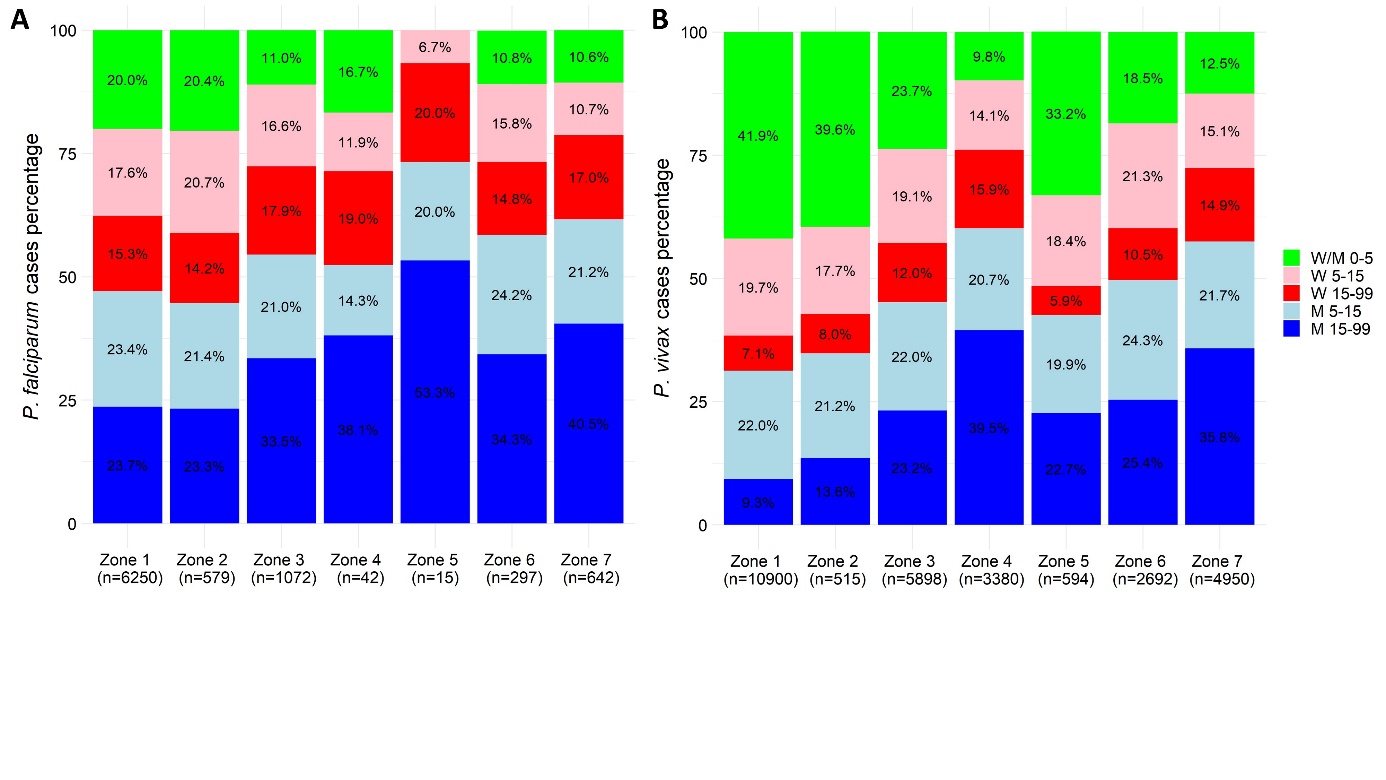


## Table S1. Eco-epidemiological zone construction. Tables presented the number of villages in each node and proportions in brackets. A) *Plasmodium falciparum* zone construction by cross analysis between conditional inference trees (CIT) results at region and township scale. B) *Plasmodium vivax* zone construction by cross analysis between CIT results at region and township scale. C) Cross analysis between *P. falciparum* and *P. vivax* (DSC: dense forest, sparse forest, cropland landscape; DSG: dense forest, sparse forest, grassland landscape; SG: sparse forest, grassland landscape; M: midland climate; SM: south midland climate; N: node).

**(A)**

|  |  | *METF region* | |  |
| --- | --- | --- | --- | --- |
|  |  | **Midland (N3)** | **Not in Midland (N2)** | **Total** |
| *Northern Township* | **≤2km DSC; Midland (N3)** | 129 (19.5) | 0 (0) | 129 (19.5) |
|  | **≤2km DSC; Foothills (N4)** | 0 (0) | 11 (1.7) | 11 (1.7) |
|  | **>2km DSC (N5)** | 92 (13.9) | 138 (20.8) | 230 (34.7) |
|  | **Not in Northern Township** | 56 (8.5) | 236 (35.6) | 292 (44.1) |
|  | **Total** | 277 (42) | 385 (58) | 662 (100) |

**(B)**

|  |  | *METF region, 15km* | | |  |
| --- | --- | --- | --- | --- | --- |
|  |  | **Midland, South Midland (N2)** | **Not in M, SM; ≤900m SG (N4)** | **Not in M, SM; >900m SG (N5)** | **Total** |
| *Northern Township, 10km* | **Not in Midland; ≤1.7km DSC (N3)** | 0 (0) | 0 (0) | 10 (1.5) | 10 (1.5) |
|  | **Not in Midland; >1.7km DSC (N4)** | 0 (0) | 5 (0.7) | 134 (20.2) | 139 (21) |
|  | **Midland; ≤2.5km DSC (N6)** | 132 (19.9) | 0 (0) | 0 (0) | 132 (19.9) |
|  | **Midland; >2.5km DSC; ≤7.5km DSG (N8)** | 82 (12.4) | 0 (0) | 0 (0) | 82 (12.4) |
|  | **Midland; >2.5km DSC; >7.5km DSG (N9)** | 7 (1.1) | 0 (0) | 0 (0) | 7 (1.1) |
|  | **Not in Northern Township** | 71 (10.7) | 19 (2.9) | 202 (30.5) | 292 (44.1) |
|  | **Total** | 292 (44.1) | 24 (3.6) | 346 (52.3) | 662 (100) |

**(C)**

| **P. falciparum**  **P. vivax** | **Not in M; >2km DSC** | **In M;**  **>2km DSC** | **Foothills; ≤2km DSC** | **In M; ≤2km DSC** |
| --- | --- | --- | --- | --- |
| **Not in M, SM; >900m SG; >1.7km DSC** | **335 (50.6)**  **Z7** | 0 (0) | 1 (0.15) | 0 (0) |
| **Not in M, SM; ≤900m SG; >1.7km DSC** | **24 (3.6)**  **Z4** | 0 (0) | 0 (0) | 0 (0) |
| **Not in M, SM; ≤1.7km DSC; >900m SG** | 0 (0) | 0 (0) | **10 (1.5)**  **Z2** | 0 (0) |
| **In M, SM; >2.5km DSC; >7.5km DSG** | **15 (2.3)**  **Z5** | **63 (9.5)**  **Z6** | 0 (0) | 0 (0) |
| **In M; ≤2.5km DSC** | 0 (0) | 3 (0.45) | 0 (0) | **129 (19.4)**  **Z1** |
| **In M; >2.5km DSC; ≤7.5km DSG** | 0 (0) | **82 (12.4)**  **Z3** | 0 (0) | 0 (0) |

1. USGS EROS Archive. USGS EROS Archive - Digital Elevation - Global Multi-resolution Terrain Elevation Data 2010 (GMTED2010). [↑](#footnote-ref-1)
2. Rutledge D. Landscape indices as measures of the effects of fragmentation : can pattern reflect process ? *DOC Sci Intern Ser 98*. 2003:1-27. [↑](#footnote-ref-2)
